# Metagenomics Reveals Seasonal Functional Adaptation of the Gut Microbiome to Host Feeding and Fasting in the Chinese Alligator

**DOI:** 10.3389/fmicb.2019.02409

**Published:** 2019-10-25

**Authors:** Ke-Yi Tang, Zhen-Wei Wang, Qiu-Hong Wan, Sheng-Guo Fang

**Affiliations:** ^1^MOE Key Laboratory of Biosystems Homeostasis & Protection, State Conservation Centre for Gene Resources of Endangered Wildlife, College of Life Sciences, Zhejiang University, Hangzhou, China; ^2^Changxing Yinjiabian Chinese Alligator Nature Reserve, Changxing, China

**Keywords:** hibernation, gut microbiota, Chinese alligator, metagenomics, mucin glycan degradation, fasting, feeding

## Abstract

As a natural hibernator, the Chinese alligator (*Alligator sinensis*) is an ideal and intriguing model to investigate changes in microbial community structure and function caused by hibernation. In this study, we used 16S rRNA profiling and metagenomic analysis to compare the composition, diversity, and functional capacity in the gut microbiome of hibernating vs. active Chinese alligators. Our results show that gut microbial communities undergo seasonal restructuring in response to seasonal cycles of feeding and fasting in the Chinese alligator, but this animal harbors a core gut microbial community primarily dominated by Proteobacteria, Fusobacteria, Bacteroidetes, and Firmicutes across the gut regions. During hibernation, there is an increase in the abundance of bacterial taxa (e.g., the genus *Bacteroides*) that can degrade host mucin glycans, which allows adaptation to winter fasting. This is accompanied by the enrichment of mucin oligosaccharide-degrading enzyme and carbohydrate-active enzyme families. In contrast, during the active phase (feeding), active Chinese alligators exhibit a carnivore gut microbiome dominated by Fusobacteria, and there is an increase in the relative abundance of bacteria (e.g., *Cetobacterium somerae*) with known proteolytic and amino acids-fermentating functions that improve host protein-rich food digestion efficiency. In addition, seasonal variations in the expression of β-defensins play a protective role in intestinal immunity. These findings provide insights into the functional adaptations of host–gut microbe symbioses to seasonal dietary shifts to maintain gut homeostasis and health, especially in extreme physiological states.

## Introduction

The animal gastrointestinal (GI) tract harbors diverse and complex microbial ecosystems that profoundly affect numerous aspects of host biology (Jandhyala et al., 2015), including nutrient extraction (Shortt et al., 2018), development of the immune system (Thaiss et al., 2016), and resistance to invading pathogens (Ouwerkerk et al., 2013). In turn, host organisms provide a favorable environment and diet- or host-derived nutrients that sustain the growth of gut microbial communities (Carey and Assadi-Porter, 2017). Most studies of the gut microbiome to date have focused on endotherms, including humans, but there are relatively few reports on gut microbiota in ectotherms. Some studies have investigated the influence of geographical location (Ahasan et al., 2018), diet (Hong et al., 2011; Xia et al., 2014; Kohl et al., 2016; Jiang et al., 2017), captivity (Kohl et al., 2017), host genetics (Yuan et al., 2015; Ren et al., 2016), and gut region (Colston et al., 2015; Abdelrhman et al., 2016; Kohl et al., 2017) on gut microbial communities in reptiles. However, there is little information about gut microbiota in Crocodilia, with only one study describing the influence of fasting on gut bacterial flora in the American alligator (Keenan et al., 2013).

The Chinese alligator (*Alligator sinensis*) is an ancient, endangered and endemic freshwater crocodilian that was listed as a first-class protected species by the Chinese government in 1972 (Thorbjarnarson and Xiaoming, 1999). Besides, it was also listed as a critically endangered species in the International Union for Conservation of Nature and Natural Resources (IUCN) Red List (Zhang et al., 2016). In recent years, wild populations have suffered a sustained decline because of habitat loss, environmental pollution, and hunting (Chen et al., 2003; Wan et al., 2013). As a semi-aquatic obligate carnivore, this ancient species feeds and forages within the freshwater, and its prey is primarily made up of protein-rich freshwater fishes (Livingstone, 2012). The Chinese alligator is a poikilotherm whose body temperature (*T*_*b*_) varies with the ambient temperature. It completely ceases food intake and body movement and enters a state of hibernation when the environmental temperature falls below 14°C (Zhang et al., 2017). Unlike mammals, the Chinese alligator is not interrupted by spontaneous periodic arousals, which are characteristic of the 13-lined ground squirrel (Stevenson et al., 2014).

Hibernation is an ideal model for examining the effects of extreme dietary changes that occur annually on gut microbial community composition and function (Dill-McFarland et al., 2014). As a hibernator, the Chinese alligator experiences hibernation periods lasting several months, which involve voluntary fasting due to food unavailability and low temperatures (Chen et al., 2003). Fasting induces shifts in gut microbial communities in penguins (Dewar et al., 2014) and Syrian hamsters (Sonoyama et al., 2009). Furthermore, hibernation has been shown to alter the composition and diversity of gut microbiota in several animals including brown bears (Sommer et al., 2016), 13-lined ground squirrels (Dill-McFarland et al., 2014), Arctic ground squirrels (Stevenson et al., 2014), Syrian hamsters (Sonoyama et al., 2009), bats (Malinicova et al., 2017), tadpoles (Kohl and Yahn, 2016), and tree frogs (Weng et al., 2016). However, direct evidence for functional variations induced by hibernation in the gut microbiome (metagenome), proteome and metabolome is not yet reported (Carey and Assadi-Porter, 2017).

During host fasting, gut microbes are presumed to degrade and utilize host-derived substrates including mucin glycans and nutrients in sloughed gut epithelia to support growth and provide energy to the host (Martens et al., 2008; Carey et al., 2013). However, there is no direct functional evidence for the utilization of host-derived nutrients by gut microbes during hibernation, and the mechanisms underlying the highly efficient mucin glycan utilization observed in the gut microbiome of hibernating animals are not fully understood. Furthermore, few studies have investigated how the seasonal dynamics of reptilian gut microbiota and host–microbiota interactions enable physiological adaptation to the absence of diet-derived nutrients.

We addressed these questions in the present study by using 16S rRNA profiling and shotgun metagenomic sequencing to investigate seasonal variations in the gut microbiome of the Chinese alligator and the functional significance thereof. We hypothesize that seasonal changes in gut microbial compositions and potential function associate with altered physiological and nutritional states in the Chinese alligator between hibernation and the active phase. We also detected seasonal expression of antimicrobial peptides genes and immune-related genes to investigate host immune response to hibernation. This ancient and endangered species can advance our understanding of the interrelationships between gut microorganisms and their host, as well as functional adaptations of gut microbiota and the intestinal immune system to the diet- and hibernation-associated changes.

## Materials and Methods

### Sample Collection

This study was carried out with permission from the State Forestry Administration of China (Forest Conservation Permission Document (2014) 1545). Biological samples were obtained from Chinese alligators at the Changxing Yin-jiabian Chinese Alligator Nature Reserve according to the guidelines and approval of the Animal Ethics Committee of Zhejiang University (ZJU2015-154-13). Hibernating Chinese alligators (*n* = 3) were dug out of their caves during hibernation (January), while active Chinese alligators (*n* = 3) were captured during their active period (July). Basic information on the six Chinese alligators analyzed in this study is shown in Supplementary Table S1. The Chinese alligators were dissected, and the gastrointestinal tracts were ligated at the junction of stomach and duodenum, and the duodenum and colon. The stomach, duodenum and colon were successively opened. The luminal stomach, duodenum, and colon contents (SC, DC, and CC, respectively) were collected separately at a super-clean bench, and fecal samples (F) were collected from the cloaca. The samples were stored at −80°C for DNA extraction. After the gut contents were removed, tissue samples from the three different sections (stomach, duodenum and colon) of the GI tracts were obtained and stored in liquid nitrogen for RNA extraction.

### DNA Extraction and 16S rRNA Gene Sequencing

Total bacterial DNA was extracted from gut contents and fecal samples using the QIAamp Fast DNA Stool Mini Kit (Qiagen, Hilden, Germany; cat. no. 51604) according to the manufacturer’s instructions and stored at −80°C until analysis. The 16S rRNA gene was amplified using the 341f/806r primer set (341f, 5′-CCTAYGGGRBGCASCAG-3′ and 806r, 5′-GGACTACNNGGGTATCTAAT-3′), which targets the V3–V4 hypervariable region of the gene. All PCRs were performed with Phusion^®^ High-Fidelity PCR Master Mix (New England, Biolabs, Ipswich, MA, United States) according to the manufacturer’s instruction. Sequencing libraries were generated using the TruSeq DNA PCR-Free Sample Preparation Kit (Illumina, San Diego, CA, United States) as recommended by the manufacturer. PCR products were sequenced on an Illumina HiSeq platform (2 × 250 bp paired-end reads) by Novogene Bioinformatics Technology Corporation (Beijing, China).

### Bioinformatics and Amplicon Sequencing Data Analyses

The overlapping paired-end reads were merged using FLASH software (Magoč and Salzberg, 2011). High-quality clean tags were obtained after quality filtering of raw tags under specific conditions according to the QIIME v.1.7.0 quality control process (Caporaso et al., 2010). Nucleotide sequences showing 97% identity in the 16S region were clustered into operational taxonomic units (OTUs) using UPARSE software (Edgar, 2013), and a representative sequence for each OTU was screened for further annotation using RDP Classifier v.2.2 (Wang et al., 2007) by searching the GreenGene Database.

Alpha diversity (i.e., Observed_species, Chao1, Shannon, Simpson, ACE, and Good-coverage) matrices and Unweighted Pair-group Method with Arithmetic Means (UPGMA) clustering were performed using QIIME and displayed using R v.3.3.3. software (Jiang et al., 2017). Beta diversity of both weighted and unweighted Unifrac was calculated using QIIME and visualized by two-dimensional principal coordinate analysis (PCoA). The diversity indices were compared among samples with the Wilcoxon rank-sum test. Analysis of similarity (ANOSIM) was performed based on the Bray–Curtis distance matrix using the R vegan package. We also compared the relative abundance of bacteria at various taxonomic levels based on the linear discriminatory analysis (LDA) effect size (LEfSe) method using LEfSe software (Segata et al., 2011). Statistically significant differences in the relative abundance of microbiota between hibernation and active-state samples at different taxonomic levels were evaluated using MetaStats (White et al., 2009). We also used Pearson’s correlation coefficient to test the correlations of microbial composition between the feces and the colon.

### Shotgun Metagenomic Sequencing, Annotation, and Statistical Analysis

Functional profiles of Chinese alligator fecal microbiomes were obtained by analyzing shotgun metagenomic sequences. Amplified libraries were generated and sequenced on the Illumina HiSeq platform (300 bp insert size) at Novogene Bioinformatics Technology Corporation (Guo et al., 2018). After quality control, host sequences identified by Basic Local Alignment Search Tool (BLAST) search of the *A. sinensis* genome were removed. Reads were quality trimmed from both ends and assembled with SOAPdenovo. MetaGeneMark v.2.10 was used to predict open reading frames (ORFs), and redundancy was removed using CD-HIT Software (Fu et al., 2012). Unigene sequence files were then used as queries in a BLAST search against the NCBI nr protein database using DIAMOND software (Buchfink et al., 2015). The lowest common ancestor (LCA) algorithm of MEGAN4 was used to sort ORF alignments into taxonomic groups with the default parameters (Huson et al., 2011). We determined significant differences in the relative abundance of microbiota in two groups of samples using MetaStats.

For functional assignment, predicted genes were searched based on the BLAST alignment against the Kyoto Encyclopedia of Genes and Genomes (KEGG) online database (Moriya et al., 2007). Carbohydrate-active enzymes (CAZymes) were annotated based on sequences predicted by BLAST using the CAZymes Analysis Toolkit (Park et al., 2010) with parameters referred to previous study (Guo et al., 2018). Heat maps, box plots, scatter plots, and taxa summary bar charts were generated using the “ggplot2” package of R software (Wickham, 2009). Canonical correspondence analysis (CCA) was used to visualize the relationship between gut microbiota and environmental factors using R vegan package (Oksanen et al., 2016). The Mann–Whitney *U-*test was performed to compare two groups of samples for relative abundances of predicted genes. The metagenome dataset and 16S rRNA sequences in this study were deposited into the NCBI Sequence Read Archive (SRA^1^) under accession number: PRJNA539906.

### Expression of Immunity-Related Genes in the GI Tract of Chinese Alligators

Total RNA was extracted from gut tissue samples using TRIzol reagent (Invitrogen, Carlsbad, CA, United States). cDNA synthesis and PCR were carried out as described in our previous study (Tang et al., 2018); primer-specific annealing temperatures are listed in Supplementary Table S2. Relative expression levels of target genes were determined with the 2^–Δ^ ^ΔCT^ method based on Ct values of triplicate reactions. The expression of immunity-related genes was compared between hibernation and active states with the Mann–Whitney *U*-test using SPSS v.20.0 (IBM, Armonk, NY, United States) after normalizing to *A. sinensis* glyceraldehyde 3-phosphate dehydrogenase (GAPDH).

## Results

### Summary of 16S rRNA Gene Sequencing

A total of 1,169,674 high-quality 16S rRNA gene reads were obtained from 18 gut content microbiota samples [the luminal contents of the stomach (SC), duodenum (DC), and colon (CC) from three hibernating and three active alligators] and six fecal (F) microbiota samples (obtained from three hibernating and three active individuals) (Supplementary Table S3), and 2,237 unique OTUs were identified and classified to at least a domain taxonomic level with 97% sequence similarity. On average, 91 and 65% of total reads were annotated at the phylum and genus levels, respectively.

### Seasonal Changes in Gut Microbial Community Composition

**FIGURE 1:**
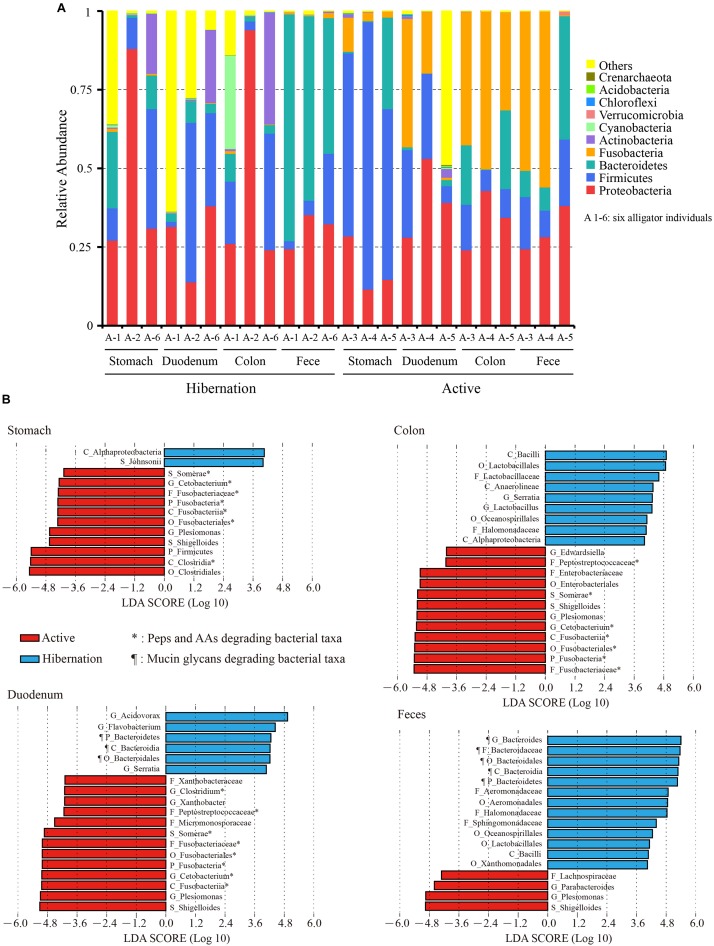
**(A)** Relative abundance of the top 10 phyla in each sample based on 16S rRNA gene sequencing. **(B)** Bacterial taxa significantly differentiated between hibernation and the active state, as determined by LEfSe. LDA scores were interpreted as the degree of difference in relative abundance. A1-6, six independent alligator individual 1–6; AAs, amino acids; C_, class; F_, family; G_, genus; O_, order; P_, phylum; Peps, peptides; S_, species.

We identified 41 bacterial phyla in the gut of the Chinese alligator by 16S rRNA gene sequencing. The most highly represented phyla (>80%) throughout the gut were Proteobacteria, Fusobacteria, Bacteroidetes, and Firmicutes (Supplementary Table S4). The top 10 most abundant phyla in each sample are shown in Figure 1A. The Chinese alligator harbored a large community of Proteobacteria (18.3–48.7%) throughout the gut during hibernation as well as in the active state. At the phylum level, microbial community composition showed seasonal variations, with the phylum Fusobacteria detected at a high level (duodenum: 20.37% vs. 0.15%, *P* = 0.04; colon: 41.22% vs. 0.39%, *P* < 0.001; feces: 35.64% vs. 0.89%, *P* = 0.04; MetaStats) from the duodenum to feces in active alligators, and the phylum Bacteroidetes being more abundant during hibernation than during the active state (feces: 57.95% vs. 18.29%, *P* = 0.02; MetaStats), particularly in the feces (Supplementary Table S4).

We also compared the top 20 most abundant bacterial genera throughout the gut during hibernation vs. in the active state (Table 1). The significance of differences in relative abundance at each taxonomic level between hibernation and active states in different gut regions was evaluated using LEfSe (Figure 1B). Within Bacteroidetes, over 98% of classified OTUs were matched to the genus *Bacteroides*, which was significantly enriched in the fecal samples from hibernating Chinese alligator (55.82% vs. 8.19%; *P* < 0.001; MetaStats). During the active phase, the most predominant genus in the hindgut was *Cetobacterium*, which belongs to the phylum Fusobacteria. Within the genus *Cetobacterium*, only one bacterial species (*Cetobacterium somerae*) was identified and was prevalent in the hindgut of active Chinese alligators (duodenum: 17.81% vs. 0.09%, *P* = 0.05; colon: 31.40% vs. 0.35%, *P* = 0.002; feces: 33.91% vs. 0.81%, *P* = 0.038; MetaStats). Proteobacteria was primarily represented by the genus *Plesiomonas*, which showed a dramatic increase in relative abundance during the active phase (stomach: 9.53% vs. 0.92%, *P* < 0.001; duodenum: 22.15% vs. 0.03%, *P* = 0.07; colon: 30.52% vs. 0.51%, *P* < 0.001; feces: 17.25% vs. 0.53%, *P* < 0.001; MetaStats).

**TABLE 1:** Comparison of relative abundance of the top 20 genera throughout the gut between hibernating and active Chinese alligator.

**Position**	**Stomach**					**Duodenum**					**Colon**					**Feces**				
**Genus**	**H**	**S.E (H)**	**A**	**S.E (A)**	***P*-value**	**H**	**S.E (H)**	**A**	**S.E (A)**	***P*-value**	**H**	**S.E (H)**	**A**	**S.E (A)**	***P*-value**	**H**	**S.E (H)**	**A**	**S.E (A)**	***P-*value**
Serratia	28.04	23.40	0.01	0.01	0.19	2.27	1.21	0.16	0.12	0.06	4.49	2.87	0.00	0.00	0.07	0.21	0.04	0.00	0.00	^∗∗^
Bacteroides	8.87	6.69	6.18	6.17	0.92	2.53	1.93	0.06	0.05	0.15	3.35	1.88	5.33	5.30	0.92	55.82	9.46	8.19	8.16	^∗∗^
Cetobacterium	0.48	0.23	4.91	2.93	0.11	0.09	0.08	20.31	11.58	**0.05**	0.35	0.20	35.82	3.16	^∗∗^	0.81	0.43	35.62	17.68	**0.04**
Plesiomonas	0.92	0.49	9.53	2.12	^∗∗^	0.03	0.01	22.15	13.64	0.07	0.51	0.36	30.52	4.99	^∗∗^	0.53	0.30	17.25	8.02	**0.01**
Clostridium	2.99	2.92	20.25	12.63	0.15	2.93	2.91	5.81	3.12	0.82	0.63	0.29	2.21	0.83	**0.04**	0.69	0.62	2.21	1.20	0.24
Bifidobacterium	6.05	6.05	0.00	0.00	0.61	7.15	7.15	0.01	0.01	0.63	11.49	11.49	0.00	0.00	0.27	0.13	0.13	0.00	0.00	0.31
Citrobacter	0.15	0.06	0.14	0.06	0.94	0.12	0.09	0.14	0.05	0.95	0.79	0.34	0.07	0.06	**0.02**	2.02	1.22	6.14	6.13	0.75
Clostridium	0.01	0.01	8.09	5.20	0.10	0.00	0.00	2.33	1.20	**0.03**	0.00	0.00	0.75	0.53	0.09	0.01	0.01	0.70	0.50	0.17
Sedimentibacter	0.39	0.33	0.02	0.01	0.21	0.16	0.16	0.03	0.02	0.76	0.57	0.38	0.02	0.02	0.09	5.02	4.98	3.49	3.45	0.90
Lactobacillus	4.04	2.38	0.04	0.02	0.08	6.06	4.51	0.31	0.27	0.15	4.08	2.12	0.01	0.01	**0.03**	0.58	0.22	0.00	0.00	**0.01**
Shewanella	0.52	0.18	0.01	0.01	**0.01**	1.77	0.78	4.62	4.62	0.84	1.71	0.55	0.01	0.01	**0.01**	0.71	0.46	0.00	0.00	0.13
Parabacteroides	0.01	0.01	3.11	3.11	0.62	0.01	0.01	0.04	0.04	0.74	0.00	0.00	1.93	1.92	0.25	0.00	0.00	3.64	3.62	0.28
Enterococcus	2.09	2.09	0.00	0.00	0.27	1.40	1.39	0.00	0.00	0.26	3.59	3.58	0.00	0.00	0.26	0.10	0.10	0.00	0.00	0.27
Escherichia	0.48	0.39	0.14	0.07	0.71	3.58	3.47	0.15	0.07	0.63	3.07	3.03	0.23	0.10	0.69	0.11	0.11	0.13	0.11	0.94
Streptococcus	1.71	1.63	0.21	0.17	0.67	2.00	1.83	0.45	0.36	0.74	3.01	2.97	0.03	0.03	0.26	0.06	0.06	0.00	0.00	0.25
Edwardsiella	0.18	0.17	0.44	0.15	0.22	0.09	0.09	2.30	1.10	**0.02**	0.08	0.07	1.72	0.72	**0.01**	1.90	1.90	2.25	1.39	0.94
Epulopiscium	0.12	0.02	1.27	0.72	0.09	0.03	0.02	0.54	0.36	0.11	0.10	0.04	1.90	1.78	0.25	0.16	0.07	3.35	1.61	**0.03**
Chelonobacter	1.63	1.16	0.03	0.02	0.13	0.37	0.35	0.01	0.01	0.24	0.05	0.02	0.00	0.00	**0.03**	0.03	0.03	0.00	0.00	0.64
Faecalibacterium	0.66	0.66	0.00	0.00	0.61	0.75	0.74	0.00	0.00	0.26	1.12	1.12	0.00	0.00	0.26	0.07	0.07	0.00	0.00	0.27
Pseudomonas	0.30	0.12	0.54	0.39	0.83	1.16	0.96	0.63	0.44	0.88	1.03	0.89	0.05	0.03	0.19	0.52	0.46	0.02	0.01	0.24

**Relative abundance were determined by 16S rRNA amplicon sequencing; numbers in bold denote a significant difference (P < 0.05). ^∗∗^P < 0.01. A, active state; H, hibernation; SE, standard error.**

### Microbial Community Diversity

**FIGURE 2:**
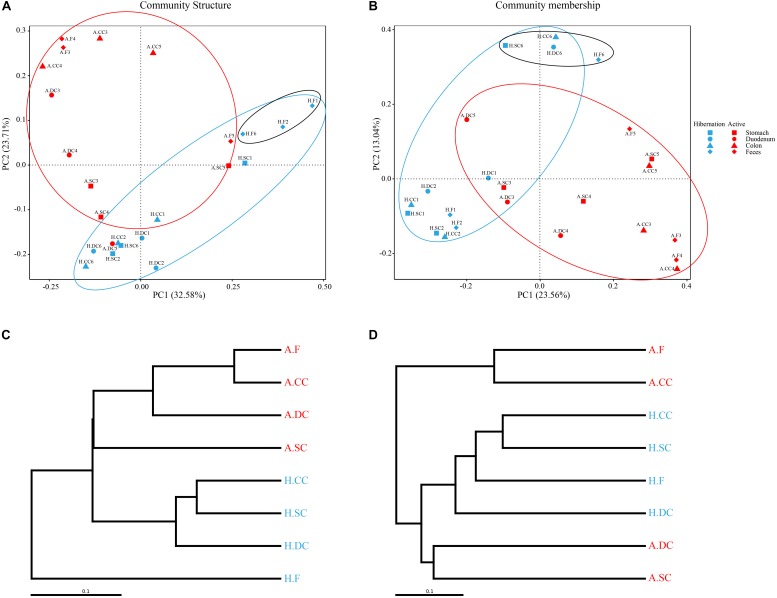
**(A,B)** Gut microbiome β-diversity during hibernation and in the active state by PCoA based on weighted **(A)** and unweighted **(B)** UniFrac distances. Each point represents the gut microbial community of an individual Chinese alligator in a given gut region. Microbial β-diversity throughout the gut was determined with UPGMA. **(C,D)** UPGMA tree of weighted **(C)** and unweighted **(D)** UniFrac distances constructed at a distance of 0.1.

We examined the α-diversity of microbiota throughout the gut sections during hibernation and in the active state based on ACE, Chao1, Observed_species, and Simpson and Shannon indices (Supplementary Table S5). Compared to active alligators, hibernating Chinese alligator had higher Chao1, ACE, and Observed_species diversity values in colonic and fecal samples (Supplementary Figure S1). There were no significant differences in the Simpson and Shannon indices between the two physiological states (Supplementary Table S6). A comparison of the α-diversity of microbes across gut regions revealed that the duodenum had the highest diversity estimates (Supplementary Figures S1, S2). As expected, PCoA of weighted and unweighted Unifrac distance matrices revealed differences in bacterial community structure (which takes into account relative abundance) and membership (presence/absence of a species) between hibernation and active states (Figures 2A,B): microbial taxa in active alligators clustered together and were separate from those in hibernating animals. We also observed a significant separation between colonic microbiota in hibernating and active alligators (weighted Unifrac distance: *P* = 0.005; Wilcoxon’s test) (Supplementary Table S7). Seasonal variations in microbial communities were further supported by UPGMA clustering of the weighted UniFrac metric (Figure 2C): in terms of community structure, hibernation samples formed a branch that was distinct from active-state samples. Moreover, colonic and fecal communities clustered together only within active samples, indicating that they had similar community structure and membership (Figures 2C,D). Pearson’s correlation analysis (Supplementary Figure S3) demonstrated a higher positive correlation between fecal and colonic microbial communities at the OTU level in the active state (*R*^2^ = 0.927, *P* < 0.001) than that during hibernation (*R*^2^ = 0.325, *P* < 0.001), underscoring the greater similarity in microbial communities of fecal and colonic samples from active as compared to hibernating animals. This result was also supported by the results of ANOSIM and the Wilcoxon test based on weighted and unweighted Unifrac distances (Supplementary Table S7).

### Summary of the Shotgun Metagenomic Datasets

For the shotgun metagenomic sequencing, we obtained 45,316 Mbp high-quality reads with an average 7 552 Mbp clean data of each sample from feces samples of six alligators (Supplementary Table S8). *De novo* assembly of feces metagenomic sequences of six Chinese alligators contained 223,756 assembled scaftigs having an average length of 1,469 bp and N50 value of 1,802 bp (Supplementary Table S9). We obtained 248,034 predicted ORFs with an average length of 727.58 bp. For taxonomic levels, 92.87 and 71.78% of the total sequences were assigned into the phylum and genus level, respectively. For functional annotation, 70.09% of the genes were classified into KEGG database; 68.99%, to eggnog; and 4.07%, to the carbohydrate-active enzymes (CAZy) database (Supplementary Table S10). Additional details of the shotgun metagenomic sequencing results are shown in Supplementary Table S10.

### Mucin Glycan Utilization by Specific Microbes for Adaptation to Fasting During Hibernation

In the present study, the phylum Bacteroidetes and genus *Bacteroides* (Figure 1B and Supplementary Figure S4), which were known mucin-utilizing bacteria, were highly represented in the microbiome of hibernating Chinese alligators. Shotgun metagenomic analysis identified 99 species within *Bacteroides* (Supplementary Table S11) of which 47 increased in relative abundance during hibernation, which was much higher than the number of species that showed increased relative abundance in the active state (*n* = 10; Supplementary Table S12). Reported host-derived mucin oligosaccharide-degrading bacteria were more highly represented during hibernation than during the active state (Supplementary Table S13). Thus, in the absence of diet-derived carbohydrates during winter fasting, the gut microbiota of the Chinese alligator appears to shift to favor bacterial species that specialize in the degradation of host-derived mucin glycans.

**FIGURE 3:**
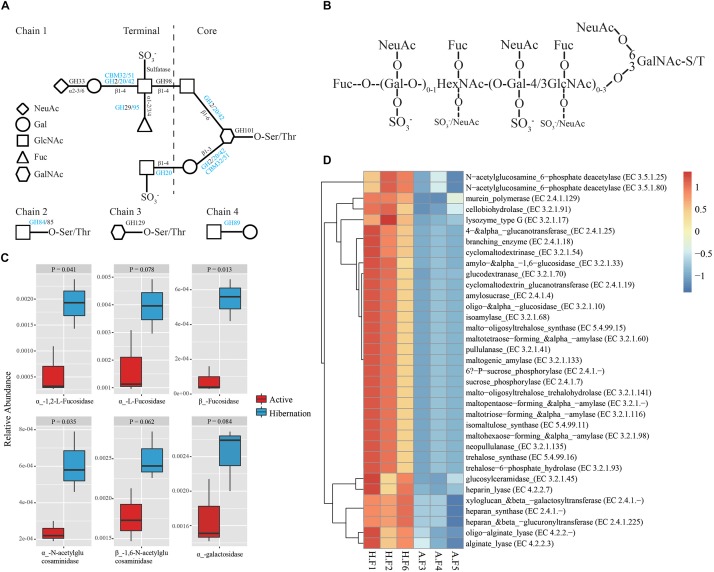
Enzymatic degradation of mucin glycans during hibernation. **(A)** Mucin glycan chains showing sites of action of GHs, CBMs, and sulfatases (Tailford et al., 2015). Chain 1 is a hypothetical mucin glycan chain; chain 2 is O-GlcNAc often present on other glycoproteins; and chains 3 and 4 are associated with gastro-duodenal mucin. Letters in blue indicate CAZyme families significantly enriched during hibernation; black indicates that there are no significant differences between the two physiological states. **(B)** Schematic composite structure of most O-linked mucin oligosaccharides (Johansson et al., 2011). The positions most often and less frequently used by different substituents are represented by large and small letters, respectively. **(C)** Differences in the relative abundance of representative mucin oligosaccharide-degrading enzymes between hibernation and the active state. Data are expressed as mean ± SE. **(D)** Heat map of the relative abundance of other CAZy enzymes showing significantly differential relative abundance between hibernation and the active state. A, active state; Fuc, fucose; F, fecal sample; Gal, galactose; GalNAc, N-acetyl-galactosamine; HexNAc, N-acetylhexosamine; H, hibernation; NeuAc, N-acetylneuraminic acid.

To further investigate the utilization of host-derived mucin glycans in the gut of hibernating Chinese alligators, we analyzed microbiome function by using the CAZy database (Vincent et al., 2014) to generate carbohydrate-active enzyme (CAZyme) profiles. In total, we identified 193 different CAZyme families comprising over 458 CAZymes. There were 30 CAZyme families with significant enrichment during hibernation (Supplementary Tables S14, S15), which was much higher than the number of CAZyme families that showed increased relative abundance in the active state (*n* = 7; Supplementary Table S14). Among CAZyme families that were significantly enriched in the hibernation group, there were 11 glycoside hydrolases (GH) families, two carbohydrate-binding modules (CBM) families, and one carbohydrate esterase (CE) family (Supplementary Table S15), some of which are known to bind and degrade various mucin oligosaccharides according to the CAZy database. In particular, CAZyme families involved in the degradation of four common mucin glycan chains were highly enriched in the microbiome of hibernating alligators (Figure 3A), including several GHs and CBMs (GH20, GH42, GH84, GH89, GH95, CBM32, and CBM51) (Supplementary Table S15).

**FIGURE 4:**
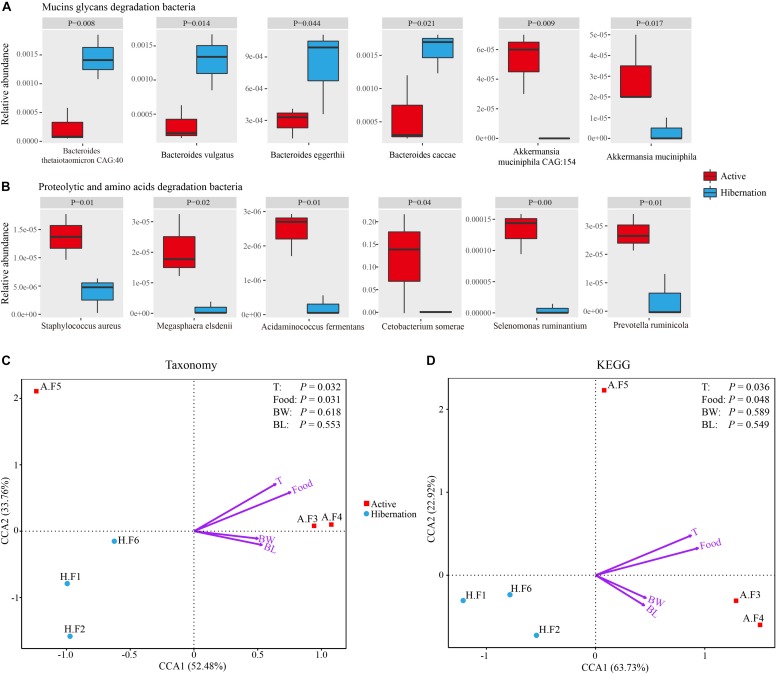
**(A,B)** Representative mucin oligosaccharide-degrading **(A)** and protein- or AA-degrading **(B)** bacterial species exhibiting significantly differential relative abundance during hibernation vs. the active state. Data are expressed as mean ± SE. **(C,D)** Relationships between environmental factors and bacterial community composition **(C)** and KEGG pathways **(D)** based on CCA. Abbreviations: BL, body length; BW, body weight; Food, food intake; F, fecal sample; T, environment temperature.

The oligosaccharides that participated in mucin glycosylation are mainly composed of one or more of four primary sugars, i.e., fucose (Fuc), galactose (Gal), N-acetylglucosamine (GlcNAc), and N-acetylgalactosamine (GalNAc) (Figures 3A,B; Derrien et al., 2004; Tailford et al., 2015). Mucins show significant structural diversity and complexity across species and gut regions, also harboring oligosaccharides such as glucose, mannose, xylose, arabinose, and other sugars (Table 2; Jensen et al., 2010; Turroni et al., 2011). Given the assortment of mucin glycosylation, we compared the relative abundance of a variety of CAZymes involved in the degradation of diverse mucin oligosaccharides between hibernation and active states (Table 2). Notably, the hibernating Chinese alligator microbiome was highly enriched in many mucin oligosaccharide-degrading enzymes (Figure 3C and Table 2). A heat map of CAZymes with significant differences in the relative abundance between the two physiological states indicated that CAZymes were enriched to a greater extent during hibernation (Figure 3D). These results demonstrate that mucin glycans are utilized as an energy source by mucin-degrading colonizers (Figure 4A) in response to food-derived nutrient unavailability during hibernation.

**TABLE 2:** Comparison of relative abundance of mucin oligosaccharide-degrading enzymes in hibernating vs. active Chinese alligators.

**Mucin oligosaccharide**	**Mucin oligosaccharide degrading enzymes**	**Relative abundance (%)**				***P*-value**
		**Hibernation**	**S.E (H)**	**Active**	**S.E (A)**	
Fucose (Fuc)	α-1,3_1,4-L-fucosidase	0.2036	0.0291	0.1158	0.0415	0.156
	α_-1,2-L-fucosidase	0.1912	0.0275	0.0563	0.0266	**0.041**
	β_-fucosidase	0.0549	0.0070	0.0079	0.0041	**0.013**
	β_-D-fucosidase	0.0105	0.0034	0.0151	0.0033	0.397
	α_-L-fucosidase	0.3948	0.0566	0.1721	0.0680	0.078
N-acetylgalactosamine (GalNAc)	α_-N-acetylgalactosaminidase	0.1419	0.0103	0.1003	0.0206	0.142
	endo-α_-N-acetylgalactosaminidase	0.0014	0.0008	0.0010	0.0006	0.706
N-acetylglucosamine (GlcNAc)	β_-1,6-N-acetylglucosaminidase	0.2503	0.0173	0.1778	0.0193	0.062
	endo-β_-N-acetylglucosaminidase	0.0794	0.0051	0.0668	0.0050	0.148
	β_-6-SO3-N-acetylglucosaminidase	0.0025	0.0002	0.0018	0.0002	0.062
	α_-N-acetylglucosaminidase	0.0610	0.0098	0.0235	0.0033	**0.035**
	N-acetylglucosamine 6-phosphate deacetylase	0.0004	0.0000	0.0003	0.0000	**0.012**
Galactose (Gal)	β_-galactosidase	0.0890	0.0040	0.0757	0.0226	0.586
	α_-galactosidase	0.2430	0.0215	0.1691	0.0229	0.084
	endo-β_-1,4-galactosidase	0.0439	0.0111	0.0324	0.0101	0.488
Glucose	α_-1,3-glucosidase	0.1064	0.0145	0.0727	0.0073	0.103
	α_-glucosidase	0.3929	0.0188	0.2887	0.0092	**0.016**
	β_-glucosidase	0.3466	0.0212	0.1865	0.0726	0.101
	amylo-β_-1,6-glucosidase	0.2213	0.0094	0.1610	0.0018	**0.011**
	oligo-α_-glucosidase	0.2204	0.0094	0.1461	0.0026	**0.006**
Mannose	β_-mannosidase	0.4478	0.0553	0.3217	0.0401	0.139
	α_-mannosidase	0.2461	0.0285	0.1892	0.0299	0.229
Arabinose	β_-L-arabinobiosidase	1.10E-06	1.83E-07	6.28E-14	1.45E-07	0.460
	α_-L-arabinosyltransferase	0.0002	0.0002	2.50E-10	9.13E-06	**0.003**
Xylose	α_-xylosidase	0.0007	0.0004	1.87E-08	7.90E-05	0.125
	β_-xylosidase	0.0005	7.95E-05	5.13E-09	4.13E-05	**0.013**
	α_-xylosyltransferase	3.11E-05	1.79E-05	2.38E-12	8.91E-07	0.424
N-acetylhexosamine (HexNAc)	β_-N-acetylhexosaminidase	0.0024	0.0002	0.0014	0.0006	0.195

**The relative abundance was determined based on CAZy database annotation; numbers in bold denote a significant difference (P < 0.05). Abbreviations: A, active state; H, hibernation; SE, standard error.**

### Carnivore Microbiomes Digest Protein-Rich Diets During the Active Phase

Given the carnivorous diet (mainly consisting of freshwater fishes) of active Chinese alligators, we compared the gut microbial composition at the phylum level of the Chinese alligator with that of known carnivores. A remarkable characteristic of the gut microbiome of carnivores is a high relative abundance of Fusobacteria (Supplementary Table S16) in the hindgut; Fusobacteria is regarded as a flesh-degrading taxon colonizing the hindgut of carnivorous animals (Roggenbuck et al., 2014). Similar to other carnivores (Supplementary Table S16), the Fusobacteria phylum, dominated by the genus *Cetobacterium* was prevalent in the GI tract of Chinese alligators in the active state. At different taxonomic levels, the microbial composition comparisons between hibernation and the active state throughout the gut revealed a higher relative abundance of proteolytic and amino acid (AA)-fermenting bacteria (e.g., *Clostridium*, Peptostreptococcaceae, and Fusobacteriaceae) in the active state (Figure 1B). A greater relative abundance of bacterial species involved in protein degradation and AA fermentation were detected in the active state by shotgun metagenomic sequencing (Supplementary Table S17 and Figure 4B), including *C. somerae*, *Selenomonas ruminantium*, *Megasphaera elsdenii*, and *Prevotella ruminicola* among others. In addition, the relative abundances of dozens of methanogenic bacteria during the active phase were higher than those during the hibernation phase, the several of them had statistically significant differences (Supplementary Table S18). Therefore, the protein-rich diet of active Chinese alligators induces a shift in the microbial community composition toward increased proteolytic and AA-metabolizing bacteria.

A search of metabolic pathways and annotated genes in the KEGG database indicated that several AA metabolism pathways (e.g., histidine metabolism) were more highly represented during feeding. At lower levels of the KEGG hierarchy, the microbiome of actively feeding alligators was enriched in genes related to D-glutamine and D-glutamate metabolism (Supplementary Figure S5A) and valine, leucine, and isoleucine biosynthesis (Supplementary Figure S5B) as compared to that of hibernating alligators. The enrichment of pathways and genes associated with AA metabolism was consistent with the increased abundance of bacteria capable of degrading and fermenting proteins and AAs in the active phase, implying that the Chinese alligator’s protein-based diet shapes the gut microbial community as an adaptation to a protein-rich diet in order to maximize nutrient extraction and energy production.

### Seasonal Immune Response and Opportunistic Intestinal Pathogens

**FIGURE 5:**
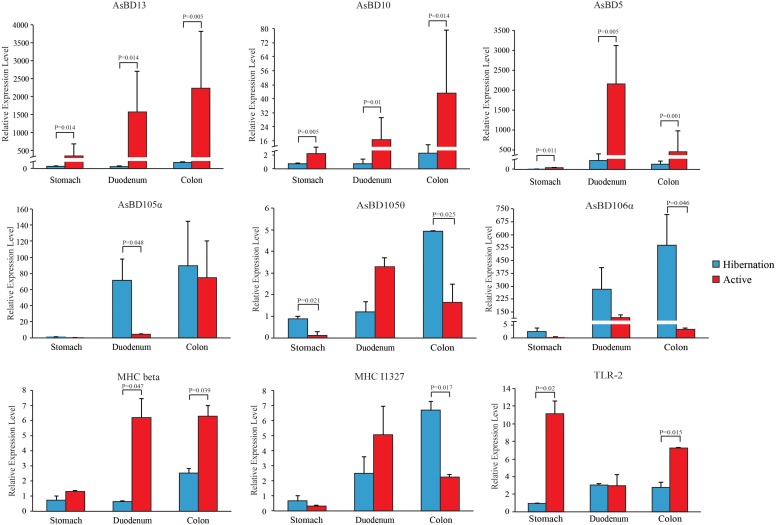
Relative expression levels of immune-related genes throughout the gut during hibernation and in the active state. Data are expressed as mean ± SE.

Given the loss of intestinal barrier function caused by mucin degradation during hibernation, we examined the effect of hibernation on the expression of immune-related genes and the relative abundance of opportunistic pathogens. In accordance with the strong expression of β-defensins in the GI tract of the Chinese alligator reported in our earlier study (Tang et al., 2018), here, we observed seasonal differences in the levels of these genes, with orthologous β-defensins (AsBD5, 10, and 13) predominating in the active state and paralogs (AsBD105α, 105θ, and 106α) being more highly expressed during hibernation (Figure 5). In addition to AsBD5 and Toll-like receptor (TLR)-2, the levels of the other seven genes increased gradually with distance along the GI tract, suggesting that immune activation is higher in the distal gut. Surprisingly, we detected viral sequences at much higher levels (4.03%) in fecal samples of active Chinese alligators than in other carnivores (Supplementary Table S19), which merits further study.

## Discussion

**FIGURE 6:**
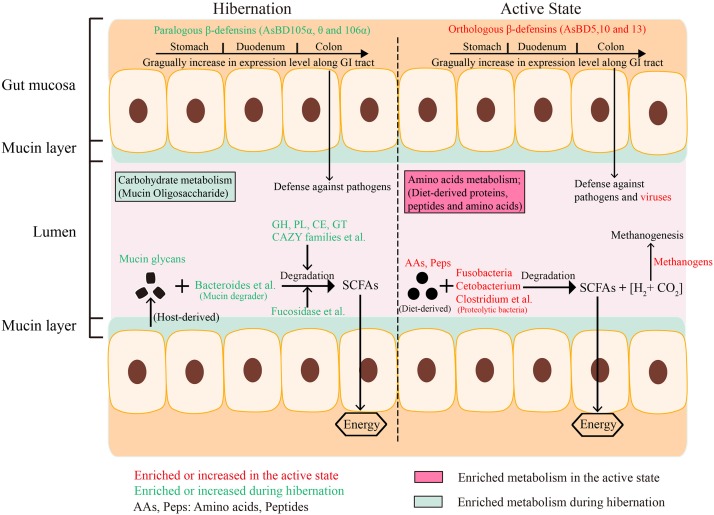
Consolidated results and overview of the effect of hibernation on gut microbial community composition, energy metabolism, enzyme enrichment, and expression of immune-related genes in the GI tract of Chinese alligator.

Previous studies have investigated the influence of hibernation on gut microbial community composition and structure based on 16S rRNA gene sequencing (Carey et al., 2013; Dill-McFarland et al., 2014; Stevenson et al., 2014), but a functional characterization of the hibernator microbiome through shotgun metagenomic profiling is lacking (Carey and Assadi-Porter, 2017). Our results highlight that the gut microbiota employs seasonal flexibility in function to degrade host-derived and diet-derived substrates during the hibernation phase and active phase, respectively, to meet their metabolic and nutritional needs (Figure 6). In particular, we further explain the molecular mechanism that enables the survival of the gut bacteria of the Chinese alligator on host-derived mucin glycan when diet-derived nutrients are absent during host hibernation.

We observed dramatic shifts in microbial community diversity and composition between hibernation and active states in the Chinese alligator, consistent with previous findings (Carey et al., 2013; Dill-McFarland et al., 2014; Sommer et al., 2016). The results of canonical correspondence analysis (CCA) revealed that the microbial taxa (Figure 4C) and functions (Figure 4D) in the fecal microbiome also showed seasonal variation. As reported earlier (Sonoyama et al., 2009; Dill-McFarland et al., 2014), dietary intake and body temperature are likely strong environmental driving forces in shaping seasonal gut microbial communities (Figures 4C,D). The predominant lineages (primarily Bacteroidetes) in the gut of hibernating alligator was more diverse at the species level (Supplementary Table S11) than those observed during the feeding state (primarily Fusobacteria and *Plesiomonas*), which may contribute to the higher diversity during hibernation. *Plesiomonas shigelloides*, which was the most abundant *Plesiomonas* species in this study, is commonly found in freshwater fish and freshwater ecosystems (Islam et al., 2011). In addition, *C. somerae* – which accounted for the majority of Fusobacteria – can inhibit the growth of other bacteria (Sugita et al., 1996), and stabilize microbial community composition when diet-derived substrates are readily available, resulting in less genetic and metabolic diversity in the active alligator gut microbiome. Captive habitat and lower food diversity (freshwater fishes account for more than 90%) may contribute to the reduction in the diversity of gut microbiota colonizing the hindgut of feeding Chinese alligator (Kohl et al., 2014; Delport et al., 2016). The gut microbiome may be used to evaluate the potential environmental adaptability of a host (Stumpf et al., 2016). Microbiome monitoring and protection of gut microbiome diversity are as important as genetic diversity in the conservation of endangered species. Different gut chambers vary in terms of pH, content, and other physiological characteristics (Kohl et al., 2017), leading to a modest dispersion of microbial communities across the gut regions even in the same physiological state (Figures 2A,B). UPGMA clustering (Figures 2C,D) and correlation analysis (Supplementary Figure S3) of colonic and fecal microbial communities in active Chinese alligators revealed that these two adjacent sections of the gut have similar microbial communities. Previous studies have used cloacal swabs for non-invasive sampling of snake gut microbiota (Colston et al., 2015) and fecal samples as a substitute for colon samples in lizards (Kohl et al., 2017). Given that collecting fecal samples is less invasive and therefore more suitable for endangered animals, we propose that fecal samples are representative of colonic communities in such species under normal physiological conditions.

Only a few microbial species from the genera *Bacteroides*, *Akkermansia*, *Ruminococcus*, and *Bifidobacterium* are known to metabolize mucins (Derrien et al., 2004; Png et al., 2010; Turroni et al., 2011; Tailford et al., 2015). Studies on hibernating squirrels (Dill-McFarland et al., 2014; Stevenson et al., 2014) have shown that hibernation is associated with increased relative abundance of known mucin-utilizing bacteria (e.g., *Bacteroides* and *Akkermansia*) with the capacity to degrade and consume host-derived mucin glycans for growth during periods of starvation or voluntary fasting (Belzer and de Vos, 2012; Foley et al., 2016). It is worth noting that Bacteroidetes was dominated by the genus *Bacteroides* in hibernating Chinese alligator, in accordance with earlier studies (Supplementary Table S16), e.g., the Burmese python, Asian sea bass, brown bear, and ground squirrel under starvation or fasting show a predominance (36–57.95%) of Bacteroidetes (Supplementary Table S16; Costello et al., 2010; Stevenson et al., 2014; Xia et al., 2014; Sommer et al., 2016). This enrichment may be explained by the ability of members of this phylum to degrade host-derived mucin glycans in the absence of dietary polysaccharides (Backhed et al., 2005; Tailford et al., 2015), allowing these bacteria to outcompete others under conditions of food shortage. Mucins are heavily glycosylated proteins composed of a core proline-threonine-serine domain that is decorated with and elongated by oligosaccharides via O- or N-linkage, with glycan accounting for up to 80% of the total mucin mass (Johansson et al., 2011; Tran et al., 2016). Based on bacterial genome sequencing, it was determined that all gut Bacteroidetes harbor polysaccharide utilization loci (PULs) that are selectively activated to metabolize diet- or host-derived glycans (Foley et al., 2016). Each PUL confers the ability to grow on a different glycan; thus, *Bacteroides* species are versatile and can cleave a variety of polysaccharides (Foley et al., 2016). For example, *B. thetaiotaomicron* encodes massive bacterial GHs and polysaccharolytic lyases (PLs) associated with degrading mucin and produces multiple fucosidases to deconstruct mucin and obtain the mucin component Fucose, resulting in high fucose availability in the GI tract (Xu et al., 2003; Sonnenburg et al., 2005; Tailford et al., 2015). *Bacteroides vulgatus* and *Bacteroides fragilis* also encode α-fucosidases for capturing L-fucose (Xu et al., 2007). In addition to Bacteroidetes, *Ruminococcus gnavus* and *Ruminococcus torques* have been reported to grow on the mucin glycans glucose, galactose, fucose, and GlcNAc as substrates (Png et al., 2010; Crost et al., 2013). The gene encoding endo-α-N-acetylgalactosaminidase and α-1, 2-L-fucosidase in *Bifidobacterium bifidum* and *Bifidobacterium longum* are highly expressed in the presence of mucin (Ruas-Madiedo et al., 2008; Turroni et al., 2011). Thus, the notable enrichment of these mucin degraders during hibernation may reflect the increased relative abundance of fucosidases and N-acetylglucosaminidases in the gut microbiome of hibernating Chinese alligator (Figures 3C, 6 and Table 2). However, only a limited number of bacterial species to date have been characterized as mucin degraders (Supplementary Table S13). It will be interesting to determine whether the many *Bacteroides* species identified in our study are capable of utilizing mucin glycans. Meanwhile, in contrast with the results obtained in the thirteen-lined ground squirrel (Stevenson et al., 2014), the decrease in the relative abundance of *Akkermansia muciniphila* during hibernation (Figure 4A) may be explained by the fact that the optimal temperature for the growth of this species is 37°C (Derrien et al., 2004), and reduced environmental temperature during hibernation is known to restrict the growth of certain microbial taxa. The CCA results demonstrate that host fasting and environmental temperature are strong forces shaping gut microbial communities in the Chinese alligator (Figures 4C,D).

The lack of diversity among endogenous GHs in vertebrate hosts (Sonnenburg et al., 2005) reflects their difficulty in utilizing mucin carbohydrates. The utilization of diverse mucin oligosaccharides depends on CAZymes encoded by mucin degraders, which convert carbohydrates into short-chain fatty acids (SCFAs), benefiting both gut-resident microbiota and the host (Foley et al., 2016; Adamberg et al., 2018). Significantly enriched CAZyme families (GH 20, GH42, GH84, GH89, GH95, CBM32, and GH51) (Supplementary Table S15) during hibernation are involved in the recognition and degradation of mucin oligosaccharides (Vincent et al., 2014). In this study we did not analyze bacterial metabolites due to the limited contents of the gut during hibernation. This further explains how Chinese alligator gut microbiota – which employs a highly organized mucin-degrading enzyme system – can subsist on enteral nutrients during the shortage of food-derived substrates under hibernation (Figure 6). The hibernation-adapted gut microbiota of the Chinese alligator is similar to human intestine-adapted bacterial symbionts, which extract energy and carbon substrates from the host under conditions of nutrient deprivation (Sonnenburg et al., 2005).

The gut microbiome of active Chinese alligators is dominated by Fusobacteria, which is also highly enriched in other carnivores (Supplementary Table S16) such as vultures (Roggenbuck et al., 2014), seals (Nelson et al., 2013), American alligators (Keenan et al., 2013), and cheetahs and jackals (Menke et al., 2014). It is associated with a protein-rich diet, suggesting that the gut microbiota of active Chinese alligators shares a carnivorous compositional feature. Accordingly, these flesh-degrading Fusobacteria exhibit proteolytic activity (Dai et al., 2011; Roggenbuck et al., 2014; Soverini et al., 2016), with optimum growth at 35–37°C and at a pH close to 7 (Olsen, 2014). Dramatic enrichment for Fusobacteria only in the gut of active Chinese alligators (except the stomach) could be explained by their carnivorous dietary regime and optimum growth conditions. This idea is consistent with results of CCA (Figure 4C), in that temperature and diet are the primary drivers for shaping the bacterial community and key members. *C. somerae*, the most abundant member of the phylum Fusobacteria in this study, is abundant in the GI tract of various fish and aquatic mammals (Larsen et al., 2014; Daniela et al., 2016; Godoy-Vitorino et al., 2017; Li et al., 2017) with a habitat and diet similar to those of Chinese alligators. This reflects their adaptation to the piscivorous diet and aquatic environment. *C. somerae* is known to ferment AAs and peptides into SCFAs that can be absorbed and utilized by both bacteria and the host (Finegold et al., 2003; Tsuchiya et al., 2010; Olsen, 2014), and it also benefits the host by producing vitamin B12 and antimicrobial peptides (Tsuchiya et al., 2010). Given that the Chinese alligator primarily feeds on freshwater fishes, we speculate that the enrichment of *C. somerae* is critical for facilitating the decomposition of animal-based proteins.

In addition to *Cetobacterium*, we also observed the enrichment of many other bacterial species (Supplementary Table S17) that are known to confer the ability to degrade and ferment proteins and AAs (Potrykus and White, 2008; Dai et al., 2010, 2011, 2013, 2015) during the active state, when the Chinese alligator has access to a protein-rich diet. Small peptides and AAs produced by these proteolytic bacteria serve as fuel for GI cells, as well as for bacteria themselves (Buckel, 2001). Moreover, fermentative and proteolytic bacteria generate SCFAs (acetate, butyrate, and propionate), ammonia, CO_2_, and H_2_ as the major end products of peptide and AA degradation and fermentation (Finegold et al., 2003; Ley et al., 2008; Tsuchiya et al., 2010). Some of these directly benefit the intestinal epithelium by providing energy, host defense, and immune regulation (Dai et al., 2011; Schwab and Gänzle, 2011; Larsen et al., 2014; Ohno, 2015). Active Chinese alligators appear to favor microbes that specialize in the degradation of protein-based substrates to potentially assist the host in enhancing energy and nutrient extraction from high-protein diets. Nevertheless, methanogenic archaea can use hydrogen and bacterial fermentation products such as acetate, formate, and methanol to reduce carbon dioxide to methane. The production of enteric methane not only aggravates global warming as a greenhouse gas, but also results in energy loss for the host (Luo et al., 2014). The significantly increased relative abundance of methanogenic bacteria in active Chinese alligators might be explained by their ability to exploit the products of bacterial AA fermentation (Supplementary Table S18), suggesting that the decrease in the efficiency of fermentative system is caused by methanogens in the gut of feeding Chinese alligator. This loss of ingested energy available to the host due to methanogenesis was also examined in pigs (Luo et al., 2014), lambs (Machmuller et al., 2000), and white rhinoceroses (Machmuller et al., 2000; Luo et al., 2013). Thus, the specific enrichment of gut proteolytic bacteria in the active state reflects the adaptation of a gut ecosystem to the host’s high-protein diet, resulting in better energy and nutrients utilization by the host and residing microbes. Overall, the seasonal variations in microbial composition and metabolic enzymes are correlated with the different nutritional requirements of the host and microbes during the fasting vs. active phase (Figure 6).

Mucin degradation is considered to be a pathogenic process since it damages the mucosal barrier, thereby increasing the permeability of the intestinal mucosa and exposing GI tract cells to harmful substances (Derrien et al., 2004; Thomas et al., 2011). Alligators are vulnerable to pathogens since they exist in bacteria-rich semi-aquatic environments (Kommanee et al., 2012). Indeed, the opportunistic intestinal pathogens of 26 genera were identified in this study, irrespective of season (Supplementary Table S20). Like reported animal pathogens, *Edwardsiella*, *Shewanella*, *Aeromonas*, *Helicobacter*, and *Enterococcus* genera, which contain species that are known opportunistic pathogens (Uddin et al., 2017; Ahasan et al., 2018; Xiang et al., 2018), were detected in the Chinese alligator gut during both seasons (Supplementary Table S20). The loss of the mucosal barrier and the presence of opportunistic pathogens are two major factors that induce the immune response in the GI tract of Chinese alligators. However, one study claimed that hibernation decreased the function of the innate and adaptive immune (Bouma et al., 2010). The intestinal immune system represents a seasonal immune response (e.g., by β-defensins) via receptors such as major histocompatibility complex (MHC) and TLR expressed on epithelial and immune cells in the GI tract of Chinese alligators. Similar to mammalian α-defensins (Tang et al., 2018), the paralogous β-defensins that are highly expressed in the GI tracts of hibernating Chinese alligators protect them against opportunistic pathogens and maintain gut symbiont homeostasis. The seasonal immune alteration of Chinese alligators is consistent with a protective immune phenotype of squirrel, which contributes to the maintenance of epithelial integrity and function during the winter fast (Kurtz and Carey, 2007). The observed gradual increase in the expression of these β-defensins along the length of the GI tract of Chinese alligators from the stomach to colon may be attributable to the parallel increase in the number of goblet cells (Chen et al., 2003) and higher concentration of pathogenic factors in the lower GI tract. The elevated levels of orthologous β-defensins, MHC-beta, and TLR2 in the gut of feeding alligators presumably reflected a protective response that may have been induced by a greater relative abundance of viruses and pathogens in the active state (Supplementary Tables S19, S20). The gut microbiome profile of captive Chinese alligators exhibits changes under increased anthropogenic pressure – such as through interactions with human keepers and the general public (Delport et al., 2016) – that can potentially lead to an increase in viral titer (from 0.04 to 4.03%) as compared to hibernating animals (Supplementary Table S19). Despite the presence of opportunistic pathogens and viruses, the robust immune response in Chinese alligator and adaptability of the gut microbial community can maintain gut homeostasis and animal health. Monitoring the Chinese alligator gut microbiome and deriving functional insights about it might provide clues to improve the environmental adaptability of this endangered species and contribute to its survival and health in extreme physiological states.

## Conclusion

Our study characterizes seasonal fluctuations and functional features in the gut microbiome of a hibernating species based on shotgun metagenomic and 16S rRNA gene sequencing. The schematic overview provides a visual abstract of the major findings but does not describe all the biochemical processes in the GI tract of Chinese alligators (Figure 6). Our results indicate that the gut microbial communities and their functional layout in Chinese alligators vary significantly between hibernation and the active state. Importantly, we showed that mucin oligosaccharide-degrading enzymes and mucin-degrading bacteria were enriched during hibernation; this allows host-derived mucin glycans to be utilized by gut bacteria, thereby supplying the host and resident microbiota with energy during food shortage. The hibernator intestinal mucosa–microbiota interactions can serve as a model for future studies on physiological states characterized by altered nutrition in the GI tract. Moreover, seasonal expression patterns of intestinal immune genes represented adaptive responses to potential pathogens and altered gut environment (e.g., loss of mucosal barrier function) induced by hibernation. Taken together, our results provide insights into the adaptive strategies employed by gut microbiota and gut immune mechanisms that contribute to the maintenance of gut ecosystem homeostasis and host health, particularly in periods of extreme dietary changes.

## Data Availability Statement

The datasets generated for this study can be found in the NCBI Sequence Read Archive under accession number: PRJNA539906.

## Ethics Statement

The animal study was reviewed and approved by the State Forestry Administration of China [Forest Conservation Permission Document (2014) 1545] and the Animal Ethics Committee of Zhejiang University (ZJU2015-154-13).

## Author Contributions

K-YT carried out the sample collection, DNA extraction, experimental procedures, data analysis, and drafted the manuscript. Z-WW participated in the sample collection. Q-HW participated in data analysis, drafting the manuscript, and study design. S-GF conceived of the study and participated in its design and coordination and helped to draft the manuscript. All authors read and approved the final manuscript.

## Conflict of Interest

The authors declare that the research was conducted in the absence of any commercial or financial relationships that could be construed as a potential conflict of interest.

## Footnotes

1 http://www.ncbi.nlm.nih.gov/sra
