# Supplementary material for: Metagenomics Reveals Seasonal Functional Adaptation of the Gut Microbiome to Host Feeding and Fasting in the Chinese Alligator
Source: Front Microbiol. 2019 Oct 25;10:2409. doi: 10.3389/fmicb.2019.02409 (PMC6824212; doi:10.3389/fmicb.2019.02409)
Supplement: Supplementary file 1 [file Table_1.DOCX]

**Supplementary materials**

**Includes:**

Five Supplemental Figures

Twenty Supplemental Tables

Supplemental Tables References

**Supplemental Figures**

**Figure S1**

**Figure** **S1.** Box-and-whisker plots for alpha-diversity estimators. (A) ACE, (B) Chao1, (C) Observed_species, and (D) Shannon indices throughout the gut of hibernating versus active Chinese alligators. Abbreviations: A, active state; CC, colon content; DC, duodenum content; F, fecal sample; H, hibernation; SC, stomach content.

**Figure S2**


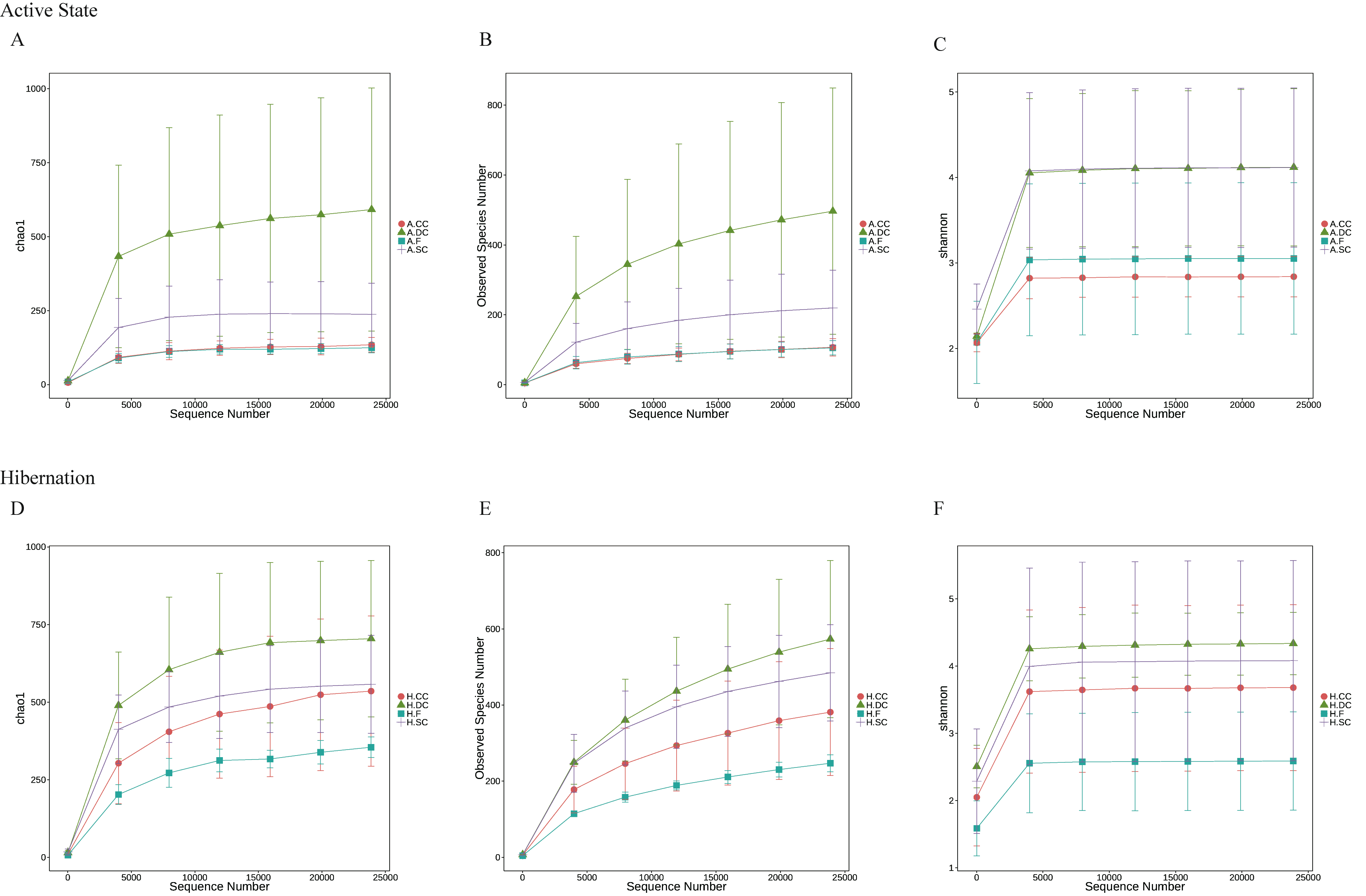


**Figure S2.** Rarefaction curves of alpha diversity indices (Chao1, Observed_species, and Shannon) in the gut of hibernating versus active Chinese alligators. Abbreviations: A, active state; CC, colon content; DC, duodenum content; F, fecal sample; H, hibernation; SC, stomach content.

**Figure S3**

**Figure S3.** Faecal microbiota composition mirrors that of the colonic lumen. Pearson’s correlations of the relative abundance of OTUs were determined between feces and colon samples.

**Figure S4**

**Figure S4.** Changes in the relative abundance of representative mucin oligosaccharide degrading bacteria (the phylum *Bacteroidetes* and genus *Bacteroides*) based on 16S rRNA sequencing and metagenomic analysis between hibernating versus active Chinese alligators. Asterisks indicate significant differences between the two physiological states. Data are expressed as mean ± SE. *P < 0.05, **P < 0.01.

**Figure S5**

**
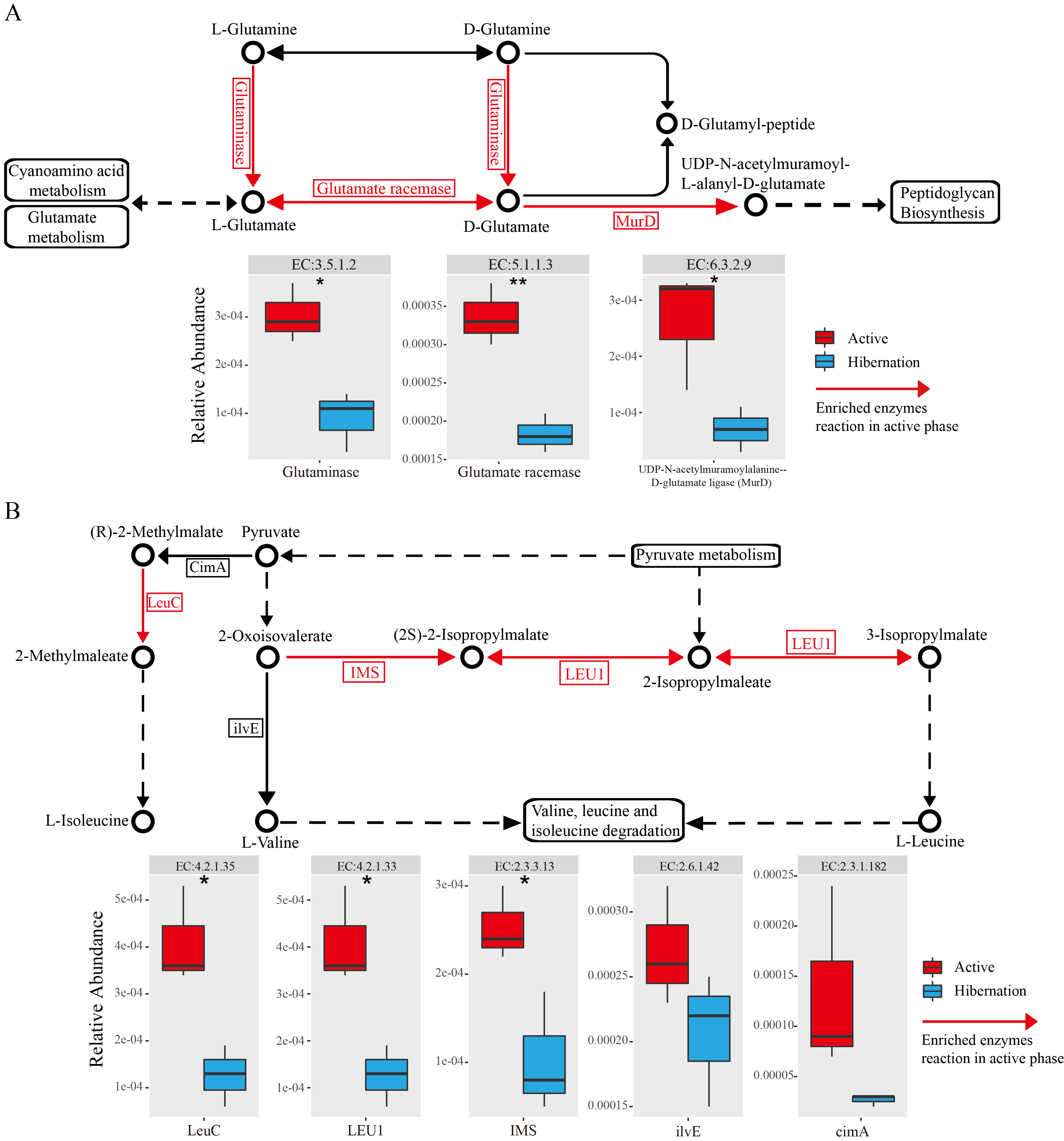
**

**Figure S5.** Highly represented AA metabolism pathways in the microbiome of active Chinese alligators. (A, B) Changes in the relative abundance of genes involved in d-glutamine and d-glutamate metabolism and valine, leucine, and isoleucine metabolism (B) are shown as box plots. Solid and dashed arrows represent one-step and multi-step processes, respectively. Asterisks indicate significant differences between hibernation and the active state. Data are expressed as mean ± SE. *P < 0.05, **P < 0.01.

**Supplemental Tables**

**Table S1.** Basic information on six Chinese alligator samples

| Individuals | Environmental Temperature/T (°C) | Food intake/Food (kg/week) | Food Source | Gender | Body Weight/BW (kg) | Body Length/BL (cm) | Group |
| --- | --- | --- | --- | --- | --- | --- | --- |
| Alligator-1 | 7.6 | 0 | − | Male | 32.5 | 180.0 | Hibernation |
| Alligator-2 | 7.2 | 0 |  | Female | 31.2 | 177.5 |  |
| Alligator-6 | 7.9 | 0 |  | Female | 24.7 | 156.5 |  |
| Alligator-3 | 32.1 | 4.5 | Freshwater Fishes | Male | 53.3 | 213.0 | Active |
| Alligator-4 | 31.9 | 4.2 |  | Female | 28.9 | 173.0 |  |
| Alligator-5 | 33.3 | 3.5 |  | Female | 25.9 | 161.1 |  |

Alligator-1, -2 and -6 were independent individual from hibernation phase, Alligator-3, -4 and -5 were independent individual from active phase. The collection of samples in this study was performed according to the permission from the State Forestry Administration of China [Forest Conservation Permission Document (2014) 1545] and the Animal Ethics Committee of Zhejiang University (ZJU2015-154-13). Chinese alligator consume predominantly freshwater fish (95%), including silver carp and grass carp, a lesser amount of crustaceans (5%), including crawfish and crabs during active phase. In summer, we feed the alligators once a week and collected gut contents of the active alligators on the third days after the last feeding. In late November, Chinese alligators enter their hibernation and stop feeding. We collected gut contents of the hibernating alligators in mid-January.

**Table S2.** Primers used for quantitative reverse transcription PCR amplification of immune-related genes

| Primer Name | Sequence (5'−3') | Ta |
| --- | --- | --- |
| qAsBD13-F | CTGCTTTCTTGCGGTGTGA | 61.0°C |
| qAsBD13-R | AGTGGCCGTGGTTGCTTCT |  |
| qAsBD10-F | GTTCCCCCACCCCATCATA | 61.0°C |
| qAsBD10-R | CTTGCCACAACGGAGCCTT |  |
| qAsBD5-F | TAGAAGTGCGGGACATCGT | 61.0°C |
| qAsBD5-R | TCCTCGCCAGTTCGTAAGC |  |
| qAsBD105α-F | GCTCCTTTTCCTTCTCTTGG | 58.4°C |
| qAsBD105α-R | CAGTGGCATCCTCTGCTTCC |  |
| qAsBD105θ-F | TGTTGCCTTCTTGGTCTCCC | 59.0°C |
| qAsBD105θ-R | GCATTGTCCTGAACCTCCTC |  |
| qAsBD106α-F | GACCCCCTGCCTTCTCTTT | 55.5°C |
| qAsBD106α-R | CCCAGCATCATCCTTCACC |  |
| MHCBeta1085-E3-F | AGGTGAAAGTCTCCCCAGC | 56.0°C |
| MHCBeta1085-E3-R | GGAAGGTCCAGTCTCCGTT |  |
| MHC I1327-E2-F | CTTGTCATAGTCGGGCACG | 59.5°C |
| MHC I1327-E2-R | CCAAACTCGCATTGAACCA |  |
| TLR-2F | TTCTTGTTCATCCTGCTCGTT | 54.6°C |
| TLR-2R | GCACTTTCTGTGGCTTCCTTT |  |
| GAPDH-F | GGAAGATGTGGCGGGATG | 60.0°C |
| GAPDH-R | TGTTGGGACACGGAATGC |  |

**Table S3.** Results of 16S rRNA gene sequencing of microbiota across the gut regions of six Chinese alligators

| Sample Name | Raw reads | Clean reads | Effective reads | AvgLen (nt) | Q20 | Q30 | GC (%) | Effective (%) | Individuals |
| --- | --- | --- | --- | --- | --- | --- | --- | --- | --- |
| H.SC1 | 45,726 | 37,366 | 24,970 | 405 | 98.53 | 97.22 | 51.81 | 54.61 | Individual-1  (Hibernation) |
| H.DC1 | 128,216 | 92,762 | 90,010 | 387 | 98.48 | 97.10 | 51.18 | 70.20 |  |
| H.CC1 | 63,803 | 51,525 | 42,269 | 410 | 98.53 | 97.20 | 53.03 | 66.25 |  |
| H.F1 | 68,803 | 54,841 | 53,810 | 423 | 98.57 | 97.29 | 49.56 | 78.21 |  |
| H.SC2 | 74,542 | 60,118 | 25,901 | 425 | 98.37 | 96.91 | 52.76 | 34.75 | Individual-2  (Hibernation) |
| H.DC2 | 83,802 | 67,611 | 57,265 | 411 | 98.23 | 96.57 | 52.01 | 68.33 |  |
| H.CC2 | 61,987 | 49,500 | 41,640 | 425 | 98.41 | 96.93 | 53.96 | 67.18 |  |
| H.F2 | 77,749 | 62,418 | 60,796 | 423 | 98.56 | 97.25 | 50.39 | 78.20 |  |
| H.SC6 | 76,173 | 48,994 | 43,931 | 416 | 97.37 | 94.67 | 53.32 | 57.67 | Individual-6  (Hibernation) |
| H.DC6 | 67,247 | 43,678 | 41,108 | 415 | 97.41 | 94.67 | 54.45 | 61.13 |  |
| H.CC6 | 74,732 | 49,826 | 47,685 | 417 | 97.43 | 94.68 | 54.63 | 63.81 |  |
| H.F6 | 63,394 | 41,476 | 38,976 | 420 | 97.59 | 95.19 | 51.21 | 61.48 |  |
| A.SC3 | 63,074 | 36,518 | 35,038 | 410 | 98.41 | 97.06 | 52.58 | 55.55 | Individual-3  (Active) |
| A.DC3 | 66,776 | 41,162 | 38,227 | 412 | 98.44 | 97.12 | 51.43 | 57.25 |  |
| A.CC3 | 61,102 | 33,520 | 31,401 | 413 | 98.40 | 97.02 | 50.4 | 51.39 |  |
| A.F3 | 76,635 | 34,343 | 31,848 | 412 | 98.38 | 97.13 | 50.62 | 41.56 |  |
| A.SC4 | 61,241 | 27,881 | 26,876 | 405 | 98.20 | 96.90 | 52.61 | 43.89 | Individual-4  (Active) |
| A.DC4 | 66,213 | 51,935 | 50,574 | 416 | 98.23 | 96.88 | 52.80 | 76.38 |  |
| A.CC4 | 77,296 | 35,363 | 33,805 | 415 | 98.38 | 97.10 | 51.46 | 43.73 |  |
| A.F4 | 75,665 | 62,391 | 58,246 | 412 | 98.30 | 97.00 | 50.55 | 76.98 |  |
| A.SC5 | 67,332 | 46,217 | 45,056 | 425 | 97.73 | 95.79 | 52.70 | 66.92 | Individual-5  (Active) |
| A.DC5 | 99,503 | 44,729 | 41,620 | 399 | 98.24 | 96.74 | 53.76 | 41.83 |  |
| A.CC5 | 60,396 | 44,296 | 41,598 | 418 | 97.93 | 96.17 | 50.82 | 68.88 |  |
| A.F5 | 71,183 | 51,204 | 32,592 | 421 | 98.01 | 96.28 | 51.32 | 45.79 |  |
| Average | 72,191 | 48,736 | 43,135 | 414 | 98.17 | 96.54 | 52.06 | 59.67 |  |

Abbreviations: A, active state; CC, colon content; DC, duodenum content; F, fecal sample; H, hibernation; SC, stomach content. Individual 1-6 are six independent Chinese alligators.

**Table S4.** Comparision of the relative abundance at the top 10 most abundant phyla between the hibernation and active phase across the gut regions of Chinese alligators. The relative abundances were obtained from 16S rRNA amplicon sequencing.

| Position | Stomach | | | Duodenum | | | Colon | | | Feces | | |
| --- | --- | --- | --- | --- | --- | --- | --- | --- | --- | --- | --- | --- |
| Phylum | Hibernation | Active | P value | Hibernation | Active | P value | Hibernation | Active | P  value | Hibernation | Active | P value |
| Proteobacteria | 48.747 | 18.284 | 0.14 | 27.847 | 40.146 | 0.20 | 48.095 | 33.816 | 0.72 | 30.694 | 30.281 | 0.90 |
| Firmicutes | 19.378 | 65.779 | **0.02** | 27.293 | 20.054 | 0.87 | 19.873 | 10.090 | 0.54 | 9.844 | 15.390 | 0.54 |
| Bacteroidetes | 11.925 | 10.008 | 0.93 | 4.189 | 0.982 | **0.03** | 4.379 | 14.712 | 0.17 | 57.954 | 18.292 | **0.02** |
| Fusobacteria | 0.542 | 5.176 | 0.13 | 0.151 | 20.370 | **0.04** | 0.394 | 41.221 | **0.00** | 0.892 | 35.639 | **0.04** |
| Actinobacteria | 6.542 | 0.380 | 0.52 | 7.948 | 1.191 | 0.62 | 12.092 | 0.075 | 0.30 | 0.168 | 0.032 | 0.36 |
| Cyanobacteria | 0.373 | 0.017 | **0.04** | 0.112 | 0.114 | 0.96 | 9.988 | 0.001 | 0.32 | 0.121 | 0 | 0.08 |
| Verrucomicrobia | 0.036 | 0.010 | 0.16 | 0.018 | 0.036 | 0.61 | 0.018 | 0 | 0.14 | 0.006 | 0.352 | 0.37 |
| Chloroflexi | 0.054 | 0.096 | 0.81 | 0.057 | 0.281 | 0.19 | 0.061 | 0.003 | 0.19 | 0.007 | 0.001 | 0.06 |
| Acidobacteria | 0.099 | 0.060 | 0.72 | 0.071 | 0.189 | 0.28 | 0.074 | 0.001 | **0.02** | 0.008 | 0 | 0.22 |
| Crenarchaeota | 0.015 | 0.008 | 0.80 | 0.014 | 0.101 | 0.60 | 0.001 | 0 | 1.00 | 0.006 | 0 | 0.16 |
| Nitrospirae | 0.077 | 0.056 | 0.84 | 0.050 | 0.081 | 0.88 | 0.034 | 0.003 | 0.12 | 0.006 | 0.001 | 0.20 |
| Others | 12.034 | 0.046 | 0.27 | 32.176 | 16.214 | 0.72 | 4.863 | 0.075 | 0.24 | 0.275 | 0.001 | 0.26 |

Numbers in bold denote a significant difference (P < 0.05).

**Table S5.** Summary of alpha diversity estimators for microbial communities during hibernation (H) and in the active (A) state throughout the gut

| Sample name |  | Richness estimates | | | Diversity estimates | |
| --- | --- | --- | --- | --- | --- | --- |
|  | Goods coverage | Observed species (Mean±SE) | Chao1 (Mean±SE) | ACE (Mean±SE) | Shannon (Mean±SE) | Simpson (Mean±SE) |
| H.SC | 0.995 | 485±127 | 557±158 | 566±146 | 4.08±1.49 | 0.75±0.22 |
| H.DC | 0.992 | 573±206 | 704±252 | 744±282 | 4.34±0.46 | 0.85±0.07 |
| H.CC | 0.994 | 381±167 | 535±242 | 555±262 | 3.68±1.23 | 0.75±0.21 |
| H.F | 0.996 | 247±22 | 355±33 | 367±44 | 2.59±0.73 | 0.63±0.14 |
| A.SC | 0.998 | 220±108 | 237±105 | 248±109 | 4.12±0.93 | 0.89±0.06 |
| A.DC | 0.995 | 497±353 | 591±411 | 600±408 | 4.12±0.92 | 0.81±0.05 |
| A.CC | 0.999 | 107±25 | 134±25 | 139±33 | 2.84±0.24 | 0.77±0.01 |
| A.F | 0.999 | 105±20 | 124±17 | 134±33 | 3.05±0.89 | 0.75±0.11 |

Abbreviations: A, active state; CC, colon content; DC, duodenum content; F, fecal sample; H, hibernation; SC, stomach content.

**Table S6.** Comparison of alpha diversity estimators between hibernation and the active state and between adjacent gut regions based on the Wilcoxon rank-sum test

| Pairwised comparison | ACE | | Chao1 | | Observed_species | | Shannon | | Simpson | | Group |
| --- | --- | --- | --- | --- | --- | --- | --- | --- | --- | --- | --- |
|  | Difference | P value | Difference | P value | Difference | P value | Difference | P value | Difference | P value |  |
| A.SC–H.SC | -8.333 | 0.061 | -7.667 | 0.076 | -8.000 | 0.053 | 1.333 | 0.819 | 4.667 | 0.449 | Same regions |
| A.DC–H.DC | -2.667 | 0.528 | -2.667 | 0.518 | -3.667 | 0.353 | -1.000 | 0.864 | -3.000 | 0.624 |  |
| A.CC–H.CC | -12.667 | **0.007** | -12.667 | **0.006** | -11.333 | **0.009** | -4.000 | 0.496 | -3.667 | 0.550 |  |
| A.F–H.F | -9.667 | **0.032** | -9.667 | **0.029** | -7.667 | 0.063 | 3.667 | 0.532 | 3.333 | 0.587 |  |
| A.SC–A.DC | 7.000 | 0.110 | 7.000 | 0.102 | 6.000 | 0.137 | -0.667 | 0.909 | -5.667 | 0.360 | Adjacent regions |
| A.DC–A.CC | -11.667 | **0.012** | -12.000 | **0.009** | -12.000 | **0.006** | -7.000 | 0.240 | -3.000 | 0.624 |  |
| A.CC–A.F | 0.333 | 0.937 | 0.667 | 0.871 | 0.667 | 0.864 | -0.667 | 0.909 | -0.333 | 0.956 |  |
| H.SC–H.DC | 1.333 | 0.751 | 2.000 | 0.627 | 1.667 | 0.670 | 1.667 | 0.775 | 2.000 | 0.744 |  |
| H.DC–H.CC | -1.667 | 0.692 | -2.000 | 0.627 | -4.333 | 0.275 | -4.000 | 0.496 | -2.333 | 0.703 |  |
| H.CC–H.F | 3.333 | 0.432 | 3.667 | 0.377 | 4.333 | 0.275 | 7.000 | 0.240 | 6.667 | 0.284 |  |

Numbers in bold denote a significant difference (P < 0.05).

**Table S7.** Comparison of microbial community similarity in the same and adjacent gut regions between hibernation and the active state by ANOSIM and with the Wilcoxon rank-sum test

| Pairwised comparison | Anosim | | Weighted_unifrac_wilcox | | Unweighted_unifrac_wilcox | | Group |
| --- | --- | --- | --- | --- | --- | --- | --- |
|  | R-value | P-value | Difference | P-value | Difference | P-value |  |
| A.SC-H.SC | 0.543 | **0.015** | -0.333 | 0.945 | 1.000 | 0.876 | Same gut regions |
| A.DC-H.DC | 0.296 | 0.30 | -7.000 | 0.159 | -1.333 | 0.835 |  |
| A.CC-H.CC | 0.778 | 0.10 | -15.333 | **0.005** | -8.000 | 0.222 |  |
| A.F-H.F | 0.593 | 0.10 | 9.333 | 0.066 | -4.333 | 0.501 |  |
| A.SC-A.DC | -0.074 | 0.80 | -3.000 | 0.536 | 0.000 | 1.000 | Adjacent gut regions |
| A.DC-A.CC | 0.111 | 0.40 | -5.333 | 0.277 | -6.333 | 0.329 |  |
| A.CC-A.F | -0.259 | 1.00 | -9.667 | 0.058 | -0.333 | 0.958 |  |
| H.SC-H.DC | 0.111 | 0.24 | 3.667 | 0.450 | 2.333 | 0.716 |  |
| H.DC-H.CC | -0.185 | 0.60 | 3.000 | 0.536 | 0.333 | 0.958 |  |
| H.CC-H.F | 0.259 | 0.10 | 15.000 | 0.006 | 3.333 | 0.604 |  |

Numbers in bold denote a significant difference (P < 0.05).

**Table S8.** Results of the shotgun metagenomic sequencing of fecal microbes in Chinese alligators

| Sample Name | Insert Size (bp) | Raw Data  (Mbp) | Raw  Reads | Clean Data(Mbp) | Q20 | Q30 | GC (%) | Effective (%) | Non-Host Data |
| --- | --- | --- | --- | --- | --- | --- | --- | --- | --- |
| H.F1 | 300 | 5,995.64 | 47,965,156 | 5,879.52 | 94.89 | 90.03 | 45.82 | 98.063 | 5,404.16 |
| H.F2 | 300 | 15,903.26 | 127,226,098 | 14,854.75 | 92.09 | 85.89 | 47.81 | 93.407 | 5,143.74 |
| H.F6 | 300 | 8,299.86 | 66,398,872 | 8,226.82 | 97.19 | 94.09 | 46.67 | 99.12 | 6,134.68 |
| A.F3 | 300 | 5,608.47 | 44,867,738 | 5,538.13 | 95.25 | 90.74 | 39.54 | 98.746 | 5,502.14 |
| A.F4 | 300 | 5,867.45 | 46,939,632 | 5,797.82 | 95.34 | 90.91 | 39.02 | 98.813 | 5,753.52 |
| A.F5 | 300 | 5,031.91 | 40,255,250 | 5,019.15 | 98.05 | 95.32 | 46.87 | 99.746 | 4,926.92 |

Abbreviations: A, active state; F, fecal sample; H, hibernation; number 1-6, six independent Chinese alligators.

**Table S9.** Scaftigs assembly results of the shotgun metagenomic sequencing of fecal microbes in Chinese alligators

| Sample Name | Total length (bp) | Average length (bp) | N50 length (bp) | N90 length (bp) |
| --- | --- | --- | --- | --- |
| H.F1 | 25,557,310 | 1,406.33 | 1,673 | 608 |
| H.F2 | 53,158,701 | 1,322.98 | 1,609 | 612 |
| H.F6 | 43,641,296 | 1,665.76 | 2,510 | 658 |
| A.F3 | 72,426,454 | 1,499.26 | 1,966 | 638 |
| A.F4 | 69,524,406 | 1,492.58 | 1,975 | 640 |
| A.F5 | 68,420,757 | 1,543.96 | 2,049 | 644 |

Abbreviations: A, active state; F, fecal sample; H, hibernation; number 1-6, six independent Chinese alligators.

**Table S10.** Summary of the shotgun metagenomic sequencing data obtained from Chinese alligator fecal samples

| **Sequencing data** | **Our results** |
| --- | --- |
| Total Raw Data (Mbp) | 46,706.59 |
| Total Clean Data (Mbp) | 45,316.19 |
| Effective percent | 97.02% |
| Average Clean Data (Mbp) | 7,552.70 |
| Total length of Scaftigs (bp) | 338,752,916 |
| Average length (bp) | 1,469 |
| N50 length (bp) | 1,802 |
| Predicted ORFs number | 248,034 |
| Complete ORFs number | 116,908 |
| Complete ORFs precent | 47.13% |
| Average length (bp) | 727.58 |
| GC percent | 44.04% |
| Annotated on NR Database | 212,567 (85.70%) |
| Annotated on Unclassified | 5.01% |
| Annotated on Phylum level | 92.87% |
| Annotated on Class level | 89.96% |
| Annotated on Order level | 89.34% |
| Annotated on Family level | 80.16% |
| Annotated on Genus level | 71.78% |
| Gene catalogue | 248,034 |
| Annotated on KEGG | 173,841 (70.09%) |
| Annotated on eggNOG | 171,113 (68.99%) |
| Annotated on CAZy | 10,104 (4.07%) |

**Table S11.** Relative abundance of bacterial species in the genus *Bacteroides* from the shotgun metagenome datasets for hibernation and the active state

| Species | Relative abundance (%) | | | | P value |
| --- | --- | --- | --- | --- | --- |
|  | Hibernation | S.E (H) | Active | S.E (A) |  |
| g__Bacteroides;s__Bacteroides fragilis | 0.023262524 | 0.004458695 | 0.047129092 | 0.043739033 | 0.640 |
| g__Bacteroides;s__Bacteroides graminisolvens | 0.010784611 | 0.001494365 | 0.002214709 | 0.000274553 | 0.005 |
| g__Bacteroides;s__Bacteroides nordii | 0.008893261 | 0.001370406 | 0.001271218 | 0.000407977 | 0.005 |
| g__Bacteroides;s__Bacteroides pyogenes | 0.003949818 | 0.000976032 | 0.001149391 | 0.000174592 | 0.021 |
| g__Bacteroides;s__Bacteroides salyersiae | 0.00391689 | 0.000649557 | 0.000470409 | 0.000120936 | 0.006 |
| g__Bacteroides;s__Bacteroides sp. HPS0048 | 0.003353972 | 0.000662567 | 0.000743629 | 0.000165407 | 0.010 |
| g__Bacteroides;s__Bacteroides fluxus | 0.002989366 | 0.000506457 | 0.001734829 | 0.001282451 | 0.513 |
| g__Bacteroides;s__Bacteroides helcogenes | 0.002952801 | 0.000581284 | 0.000761621 | 0.000104404 | 0.011 |
| g__Bacteroides;s__Bacteroides ovatus | 0.002926589 | 0.000399658 | 0.000742942 | 0.000368094 | 0.010 |
| g__Bacteroides;s__Bacteroides oleiciplenus | 0.002919808 | 0.000393839 | 0.000951616 | 0.00016215 | 0.007 |
| g__Bacteroides;s__Bacteroides faecichinchillae | 0.002637627 | 0.000376563 | 0.00036986 | 0.000126937 | 0.005 |
| g__Bacteroides;s__Bacteroides stercorirosoris | 0.002375365 | 0.000164865 | 0.000398725 | 0.000155847 | 0.002 |
| g__Bacteroides;s__Bacteroides reticulotermitis | 0.002314161 | 0.000185039 | 0.000512371 | 4.23E-05 | 0.001 |
| g__Bacteroides;s__Bacteroides cellulosilyticus | 0.002228882 | 0.000400599 | 0.000471728 | 0.000147718 | 0.009 |
| g__Bacteroides;s__Bacteroides finegoldii | 0.002167285 | 0.000424092 | 0.000237057 | 3.13E-05 | 0.007 |
| g__Bacteroides;s__Bacteroides sp. CAG:754 | 0.001894641 | 0.000318013 | 0.000485915 | 7.96E-05 | 0.008 |
| g__Bacteroides;s__Bacteroides sp. D2 | 0.001833342 | 0.000426459 | 0.000519627 | 6.88E-05 | 0.018 |
| g__Bacteroides;s__Bacteroides acidifaciens | 0.00178264 | 0.000168849 | 0.000272124 | 0.00011526 | 0.003 |
| g__Bacteroides;s__Bacteroides caccae | 0.00157933 | 0.000178041 | 0.000582705 | 0.000310186 | 0.021 |
| g__Bacteroides;s__Bacteroides sp. CAG:633 | 0.001483648 | 0.000356881 | 0.000580646 | 0.000217516 | 0.044 |
| g__Bacteroides;s__Bacteroides thetaiotaomicron CAG:40 | 0.001444298 | 0.000224925 | 0.000239089 | 0.000172627 | 0.008 |
| g__Bacteroides;s__Bacteroides vulgatus | 0.0012886 | 0.000238754 | 0.00033498 | 0.000149559 | 0.014 |
| g__Bacteroides;s__Bacteroides coprosuis | 0.001269434 | 0.000216223 | 0.000584034 | 0.000220122 | 0.040 |
| g__Bacteroides;s__Bacteroides thetaiotaomicron | 0.001246858 | 0.000189172 | 0.000710148 | 0.000458751 | 0.321 |
| g__Bacteroides;s__Bacteroides gallinarum | 0.001170154 | 0.000211673 | 0.000292592 | 3.26E-05 | 0.009 |
| g__Bacteroides;s__Bacteroides intestinalis CAG:315 | 0.001165528 | 0.000342472 | 0.000132299 | 3.54E-05 | 0.018 |
| g__Bacteroides;s__Bacteroides sp. CAG:530 | 0.001033175 | 0.000243549 | 0.000350119 | 0.000104041 | 0.026 |
| g__Bacteroides;s__Bacteroides uniformis | 0.000987024 | 9.69E-05 | 0.000864598 | 0.000463982 | 0.788 |
| g__Bacteroides;s__Bacteroides intestinalis | 0.000919428 | 0.000194951 | 0.000156556 | 6.56E-05 | 0.011 |
| g__Bacteroides;s__Bacteroides eggerthii | 0.00082029 | 0.00023105 | 0.000291155 | 8.20E-05 | 0.044 |
| g__Bacteroides;s__Bacteroides sp. 3_1_19 | 0.000813279 | 0.00028476 | 0.000203005 | 0.000122549 | 0.090 |
| g__Bacteroides;s__Bacteroides sp. CAG:598 | 0.000736549 | 0.000115313 | 0.000243842 | 7.82E-05 | 0.012 |
| g__Bacteroides;s__Bacteroides sp. 14(A) | 0.000736345 | 0.000259189 | 0.00012806 | 5.33E-05 | 0.035 |
| g__Bacteroides;s__Bacteroides dorei | 0.000704798 | 3.44E-05 | 0.000296223 | 6.80E-05 | 0.005 |
| g__Bacteroides;s__Bacteroides sp. 2_2_4 | 0.000676038 | 7.57E-05 | 0.00011385 | 1.44E-05 | 0.003 |
| g__Bacteroides;s__Bacteroides clarus | 0.000675642 | 0.000211192 | 0.000141813 | 6.63E-05 | 0.031 |
| g__Bacteroides;s__Bacteroides massiliensis | 0.000659885 | 3.61E-05 | 0.000438056 | 0.000126062 | 0.194 |
| g__Bacteroides;s__Bacteroides sp. CAG:661 | 0.000640143 | 0.000141219 | 0.000138717 | 1.17E-05 | 0.012 |
| g__Bacteroides;s__Bacteroides sp. 3_1_23 | 0.000561132 | 0.000141644 | 0.000139689 | 1.48E-05 | 0.019 |
| g__Bacteroides;s__Bacteroides sp. CAG:98 | 0.000540428 | 5.16E-05 | 0.000178677 | 1.50E-05 | 0.003 |
| g__Bacteroides;s__Bacteroides plebeius CAG:211 | 0.000527198 | 3.97E-05 | 7.24E-05 | 2.93E-05 | 0.001 |
| g__Bacteroides;s__Bacteroides stercoris | 0.000466133 | 0.000151387 | 0.000290617 | 7.53E-05 | 0.339 |
| g__Bacteroides;s__Bacteroides clarus CAG:160 | 0.000450104 | 7.89E-05 | 3.07E-05 | 2.52E-06 | 0.006 |
| g__Bacteroides;s__Bacteroides paurosaccharolyticus | 0.000416101 | 8.05E-05 | 0.00050681 | 4.33E-05 | 0.461 |
| g__Bacteroides;s__Bacteroides fragilis CAG:558 | 0.000396674 | 7.17E-05 | 0.001735787 | 0.001646799 | 0.550 |
| g__Bacteroides;s__Bacteroides sartorii | 0.000377093 | 0.000105361 | 9.32E-05 | 2.36E-05 | 0.025 |
| g__Bacteroides;s__Bacteroides sp. 2_1_33B | 0.000366841 | 3.59E-05 | 0.00016291 | 3.08E-05 | 0.008 |
| g__Bacteroides;s__Bacteroides sp. CAG:462 | 0.000365305 | 3.02E-05 | 0.000255794 | 8.23E-05 | 0.276 |
| g__Bacteroides;s__Bacteroides sp. CAG:927 | 0.000353174 | 8.11E-05 | 0.000247011 | 5.42E-05 | 0.319 |
| g__Bacteroides;s__Bacteroides uniformis CAG:3 | 0.000343913 | 0.000131857 | 2.32E-05 | 7.45E-06 | 0.031 |
| g__Bacteroides;s__Bacteroides sp. 1_1_14 | 0.00032436 | 0.000157297 | 0.00014669 | 0.000135129 | 0.534 |
| g__Bacteroides;s__Bacteroides faecis CAG:32 | 0.000312445 | 6.03E-05 | 3.33E-05 | 2.87E-05 | 0.009 |
| g__Bacteroides;s__Bacteroides faecis | 0.000309581 | 6.90E-05 | 7.50E-05 | 4.04E-05 | 0.019 |
| g__Bacteroides;s__Bacteroides xylanisolvens | 0.000297285 | 8.37E-05 | 0.000157316 | 9.33E-06 | 0.201 |
| g__Bacteroides;s__Bacteroides eggerthii CAG:109 | 0.000293851 | 6.36E-06 | 0.000635201 | 6.20E-05 | 0.005 |
| g__Bacteroides;s__Bacteroides sp. CAG:875 | 0.000286922 | 3.48E-05 | 0.000151625 | 2.12E-05 | 0.014 |
| g__Bacteroides;s__Bacteroides sp. D22 | 0.000263685 | 7.59E-05 | 1.74E-05 | 4.42E-06 | 0.015 |
| g__Bacteroides;s__Bacteroides stercoris CAG:120 | 0.000251659 | 2.90E-05 | 2.28E-05 | 3.41E-06 | 0.002 |
| g__Bacteroides;s__Bacteroides sp. 3_1_33FAA | 0.000243179 | 5.21E-05 | 0.000126491 | 8.48E-05 | 0.294 |
| g__Bacteroides;s__Bacteroides sp. D1 | 0.000234704 | 4.83E-05 | 9.37E-06 | 9.08E-06 | 0.007 |
| g__Bacteroides;s__Bacteroides sp. CAG:189 | 0.000227988 | 6.94E-05 | 8.67E-05 | 1.03E-05 | 0.059 |
| g__Bacteroides;s__Bacteroides sp. CAG:714 | 0.000208855 | 5.07E-05 | 0.000297973 | 2.58E-05 | 0.219 |
| g__Bacteroides;s__Bacteroides rodentium | 0.000194618 | 3.60E-05 | 0.00020851 | 3.55E-05 | 0.778 |
| g__Bacteroides;s__Bacteroides sp. 4_1_36 | 0.00018966 | 6.74E-05 | 2.26E-06 | 6.46E-07 | 0.022 |
| g__Bacteroides;s__Bacteroides salanitronis | 0.000163674 | 3.17E-05 | 0.000126021 | 5.54E-05 | 0.625 |
| g__Bacteroides;s__Bacteroides sp. 1_1_6 | 0.000152822 | 7.96E-05 | 0.000141396 | 6.61E-05 | 0.905 |
| g__Bacteroides;s__Bacteroides coprocola | 0.000147434 | 1.87E-05 | 0.000103292 | 7.58E-06 | 0.041 |
| g__Bacteroides;s__Bacteroides sp. CAG:702 | 0.000141911 | 5.59E-05 | 0.00012446 | 3.48E-05 | 0.785 |
| g__Bacteroides;s__Bacteroides sp. CAG:443 | 8.99E-05 | 3.42E-05 | 0.00016294 | 4.51E-05 | 0.267 |
| g__Bacteroides;s__Bacteroides coprocola CAG:162 | 8.64E-05 | 7.13E-06 | 0.000140485 | 3.16E-05 | 0.198 |
| g__Bacteroides;s__Bacteroides sp. CAG:144 | 7.42E-05 | 3.99E-05 | 0.001035774 | 0.00048065 | 0.071 |
| g__Bacteroides;s__Bacteroides propionicifaciens | 6.09E-05 | 9.20E-06 | 0.000176024 | 3.62E-05 | 0.017 |
| g__Bacteroides;s__Bacteroides plebeius | 6.03E-05 | 2.99E-05 | 0.000221489 | 2.20E-05 | 0.008 |
| g__Bacteroides;s__Bacteroides caccae CAG:21 | 5.10E-05 | 2.65E-05 | 0.000302479 | 0.000188326 | 0.260 |
| g__Bacteroides;s__Bacteroides sp. D20 | 4.98E-05 | 2.62E-05 | 3.36E-05 | 1.34E-05 | 0.637 |
| g__Bacteroides;s__Bacteroides sp. CAG:1076 | 3.82E-05 | 3.03E-05 | 0.000208545 | 9.14E-05 | 0.175 |
| g__Bacteroides;s__Bacteroides sp. 4_3_47FAA | 3.41E-05 | 1.73E-05 | 6.59E-06 | 6.59E-06 | 0.233 |
| g__Bacteroides;s__Bacteroides coprophilus | 3.07E-05 | 1.57E-05 | 0.000210398 | 0.00010583 | 0.197 |
| g__Bacteroides;s__Bacteroides ovatus CAG:22 | 2.82E-05 | 2.82E-05 | 8.95E-06 | 2.38E-06 | 0.593 |
| g__Bacteroides;s__Bacteroides sp. 2_1_56FAA | 2.72E-05 | 1.57E-05 | 0.00014371 | 0.000135581 | 0.536 |
| g__Bacteroides;s__Bacteroides sp. 1_1_30 | 2.55E-05 | 1.41E-05 | 0.000120348 | 6.40E-05 | 0.239 |
| g__Bacteroides;s__Bacteroides coprophilus CAG:333 | 1.51E-05 | 7.83E-06 | 0.000176567 | 1.91E-05 | 0.002 |
| g__Bacteroides;s__Bacteroides sp. CAG:20 | 1.38E-05 | 7.41E-06 | 0.000223355 | 0.000112075 | 0.144 |
| g__Bacteroides;s__Bacteroides dorei CAG:222 | 1.24E-05 | 6.52E-06 | 2.13E-07 | 1.75E-07 | 0.144 |
| g__Bacteroides;s__Bacteroides sp. 2_1_16 | 1.08E-05 | 1.07E-05 | 4.76E-05 | 4.76E-05 | 0.570 |
| g__Bacteroides;s__Bacteroides cellulosilyticus CAG:158 | 8.78E-06 | 4.88E-06 | 1.18E-05 | 3.31E-06 | 0.648 |
| g__Bacteroides;s__Bacteroides barnesiae | 6.88E-06 | 4.49E-06 | 3.13E-05 | 1.37E-06 | 0.006 |
| g__Bacteroides;s__Bacteroides sp. CAG:545 | 5.75E-06 | 4.34E-06 | 2.08E-05 | 5.44E-06 | 0.043 |
| g__Bacteroides;s__Bacteroides sp. CAG:709 | 4.70E-06 | 3.67E-06 | 1.44E-05 | 7.01E-06 | 0.281 |
| g__Bacteroides;s__Bacteroides sp. CAG:770 | 3.69E-06 | 3.37E-06 | 4.07E-05 | 2.87E-06 | 0.002 |
| g__Bacteroides;s__Bacteroides sp. CAG:1060 | 2.42E-06 | 2.42E-06 | 0 | 0 | 0.374 |
| g__Bacteroides;s__Bacteroides intestinalis CAG:564 | 1.90E-06 | 1.36E-06 | 1.38E-05 | 5.52E-06 | 0.049 |
| g__Bacteroides;s__Bacteroides finegoldii CAG:203 | 1.54E-06 | 8.40E-07 | 1.53E-05 | 3.03E-06 | 0.008 |
| g__Bacteroides;s__Bacteroides sp. 9_1_42FAA | 7.93E-07 | 3.72E-07 | 6.12E-06 | 3.10E-06 | 0.191 |
| g__Bacteroides;s__uncultured Bacteroides sp. SMG1 | 5.53E-07 | 5.53E-07 | 3.24E-06 | 7.13E-07 | 0.019 |
| g__Bacteroides;s__Bacteroides sp. 2_1_22 | 8.81E-08 | 8.81E-08 | 2.46E-06 | 1.37E-06 | 0.186 |
| g__Bacteroides;s__Bacteroides fragilis CAG:47 | 0 | 0 | 8.65E-05 | 8.65E-05 | 0.374 |
| g__Bacteroides;s__Bacteroides sp. 3_2_5 | 0 | 0 | 3.12E-06 | 3.12E-06 | 0.374 |
| g__Bacteroides;s__Bacteroides vulgatus CAG:6 | 0 | 0 | 1.77E-06 | 1.77E-06 | 0.374 |

**Table S12.** Bacterial species in the genus *Bacteroides* showing significantly differential enrichment during hibernation versus in the active state based on the shotgun metagenomic datasets

| Species | Relative abundance (%) | | | | P value |
| --- | --- | --- | --- | --- | --- |
|  | Hibernation | S.E (H) | Active | S.E (A) |  |
| g__Bacteroides;s__Bacteroides graminisolvens | 0.010784611 | 0.001494365 | 0.002214709 | 0.000274553 | 0.005 |
| g__Bacteroides;s__Bacteroides nordii | 0.008893261 | 0.001370406 | 0.001271218 | 0.000407977 | 0.005 |
| g__Bacteroides;s__Bacteroides pyogenes | 0.003949818 | 0.000976032 | 0.001149391 | 0.000174592 | 0.021 |
| g__Bacteroides;s__Bacteroides salyersiae | 0.00391689 | 0.000649557 | 0.000470409 | 0.000120936 | 0.006 |
| g__Bacteroides;s__Bacteroides sp. HPS0048 | 0.003353972 | 0.000662567 | 0.000743629 | 0.000165407 | 0.010 |
| g__Bacteroides;s__Bacteroides helcogenes | 0.002952801 | 0.000581284 | 0.000761621 | 0.000104404 | 0.011 |
| g__Bacteroides;s__Bacteroides ovatus | 0.002926589 | 0.000399658 | 0.000742942 | 0.000368094 | 0.010 |
| g__Bacteroides;s__Bacteroides oleiciplenus | 0.002919808 | 0.000393839 | 0.000951616 | 0.00016215 | 0.007 |
| g__Bacteroides;s__Bacteroides faecichinchillae | 0.002637627 | 0.000376563 | 0.00036986 | 0.000126937 | 0.005 |
| g__Bacteroides;s__Bacteroides stercorirosoris | 0.002375365 | 0.000164865 | 0.000398725 | 0.000155847 | 0.002 |
| g__Bacteroides;s__Bacteroides reticulotermitis | 0.002314161 | 0.000185039 | 0.000512371 | 4.23E-05 | 0.001 |
| g__Bacteroides;s__Bacteroides cellulosilyticus | 0.002228882 | 0.000400599 | 0.000471728 | 0.000147718 | 0.009 |
| g__Bacteroides;s__Bacteroides finegoldii | 0.002167285 | 0.000424092 | 0.000237057 | 3.13E-05 | 0.007 |
| g__Bacteroides;s__Bacteroides sp. CAG:754 | 0.001894641 | 0.000318013 | 0.000485915 | 7.96E-05 | 0.008 |
| g__Bacteroides;s__Bacteroides sp. D2 | 0.001833342 | 0.000426459 | 0.000519627 | 6.88E-05 | 0.018 |
| g__Bacteroides;s__Bacteroides acidifaciens | 0.00178264 | 0.000168849 | 0.000272124 | 0.00011526 | 0.003 |
| g__Bacteroides;s__Bacteroides caccae | 0.00157933 | 0.000178041 | 0.000582705 | 0.000310186 | 0.021 |
| g__Bacteroides;s__Bacteroides sp. CAG:633 | 0.001483648 | 0.000356881 | 0.000580646 | 0.000217516 | 0.044 |
| g__Bacteroides;s__Bacteroides thetaiotaomicron CAG:40 | 0.001444298 | 0.000224925 | 0.000239089 | 0.000172627 | 0.008 |
| g__Bacteroides;s__Bacteroides vulgatus | 0.0012886 | 0.000238754 | 0.00033498 | 0.000149559 | 0.014 |
| g__Bacteroides;s__Bacteroides coprosuis | 0.001269434 | 0.000216223 | 0.000584034 | 0.000220122 | 0.040 |
| g__Bacteroides;s__Bacteroides gallinarum | 0.001170154 | 0.000211673 | 0.000292592 | 3.26E-05 | 0.009 |
| g__Bacteroides;s__Bacteroides intestinalis CAG:315 | 0.001165528 | 0.000342472 | 0.000132299 | 3.54E-05 | 0.018 |
| g__Bacteroides;s__Bacteroides sp. CAG:530 | 0.001033175 | 0.000243549 | 0.000350119 | 0.000104041 | 0.026 |
| g__Bacteroides;s__Bacteroides intestinalis | 0.000919428 | 0.000194951 | 0.000156556 | 6.56E-05 | 0.011 |
| g__Bacteroides;s__Bacteroides eggerthii | 0.00082029 | 0.00023105 | 0.000291155 | 8.20E-05 | 0.044 |
| g__Bacteroides;s__Bacteroides sp. CAG:598 | 0.000736549 | 0.000115313 | 0.000243842 | 7.82E-05 | 0.012 |
| g__Bacteroides;s__Bacteroides sp. 14(A) | 0.000736345 | 0.000259189 | 0.00012806 | 5.33E-05 | 0.035 |
| g__Bacteroides;s__Bacteroides dorei | 0.000704798 | 3.44E-05 | 0.000296223 | 6.80E-05 | 0.005 |
| g__Bacteroides;s__Bacteroides sp. 2_2_4 | 0.000676038 | 7.57E-05 | 0.00011385 | 1.44E-05 | 0.003 |
| g__Bacteroides;s__Bacteroides clarus | 0.000675642 | 0.000211192 | 0.000141813 | 6.63E-05 | 0.031 |
| g__Bacteroides;s__Bacteroides sp. CAG:661 | 0.000640143 | 0.000141219 | 0.000138717 | 1.17E-05 | 0.012 |
| g__Bacteroides;s__Bacteroides sp. 3_1_23 | 0.000561132 | 0.000141644 | 0.000139689 | 1.48E-05 | 0.019 |
| g__Bacteroides;s__Bacteroides sp. CAG:98 | 0.000540428 | 5.16E-05 | 0.000178677 | 1.50E-05 | 0.003 |
| g__Bacteroides;s__Bacteroides plebeius CAG:211 | 0.000527198 | 3.97E-05 | 7.24E-05 | 2.93E-05 | 0.001 |
| g__Bacteroides;s__Bacteroides clarus CAG:160 | 0.000450104 | 7.89E-05 | 3.07E-05 | 2.52E-06 | 0.006 |
| g__Bacteroides;s__Bacteroides sartorii | 0.000377093 | 0.000105361 | 9.32E-05 | 2.36E-05 | 0.025 |
| g__Bacteroides;s__Bacteroides sp. 2_1_33B | 0.000366841 | 3.59E-05 | 0.00016291 | 3.08E-05 | 0.008 |
| g__Bacteroides;s__Bacteroides uniformis CAG:3 | 0.000343913 | 0.000131857 | 2.32E-05 | 7.45E-06 | 0.031 |
| g__Bacteroides;s__Bacteroides faecis CAG:32 | 0.000312445 | 6.03E-05 | 3.33E-05 | 2.87E-05 | 0.009 |
| g__Bacteroides;s__Bacteroides faecis | 0.000309581 | 6.90E-05 | 7.50E-05 | 4.04E-05 | 0.019 |
| g__Bacteroides;s__Bacteroides eggerthii CAG:109 | 0.000293851 | 6.36E-06 | 0.000635201 | 6.20E-05 | 0.005 |
| g__Bacteroides;s__Bacteroides sp. CAG:875 | 0.000286922 | 3.48E-05 | 0.000151625 | 2.12E-05 | 0.014 |
| g__Bacteroides;s__Bacteroides sp. D22 | 0.000263685 | 7.59E-05 | 1.74E-05 | 4.42E-06 | 0.015 |
| g__Bacteroides;s__Bacteroides stercoris CAG:120 | 0.000251659 | 2.90E-05 | 2.28E-05 | 3.41E-06 | 0.002 |
| g__Bacteroides;s__Bacteroides sp. D1 | 0.000234704 | 4.83E-05 | 9.37E-06 | 9.08E-06 | 0.007 |
| g__Bacteroides;s__Bacteroides sp. 4_1_36 | 0.00018966 | 6.74E-05 | 2.26E-06 | 6.46E-07 | 0.022 |
| g__Bacteroides;s__Bacteroides coprocola | 0.000147434 | 1.87E-05 | 0.000103292 | 7.58E-06 | 0.041 |
| g__Bacteroides;s__Bacteroides propionicifaciens | 6.09E-05 | 9.20E-06 | 0.000176024 | 3.62E-05 | 0.017 |
| g__Bacteroides;s__Bacteroides plebeius | 6.03E-05 | 2.99E-05 | 0.000221489 | 2.20E-05 | 0.008 |
| g__Bacteroides;s__Bacteroides coprophilus CAG:333 | 1.51E-05 | 7.83E-06 | 0.000176567 | 1.91E-05 | 0.002 |
| g__Bacteroides;s__Bacteroides barnesiae | 6.88E-06 | 4.49E-06 | 3.13E-05 | 1.37E-06 | 0.006 |
| g__Bacteroides;s__Bacteroides sp. CAG:545 | 5.75E-06 | 4.34E-06 | 2.08E-05 | 5.44E-06 | 0.043 |
| g__Bacteroides;s__Bacteroides sp. CAG:770 | 3.69E-06 | 3.37E-06 | 4.07E-05 | 2.87E-06 | 0.002 |
| g__Bacteroides;s__Bacteroides intestinalis CAG:564 | 1.90E-06 | 1.36E-06 | 1.38E-05 | 5.52E-06 | 0.049 |
| g__Bacteroides;s__Bacteroides finegoldii CAG:203 | 1.54E-06 | 8.40E-07 | 1.53E-05 | 3.03E-06 | 0.008 |
| g__Bacteroides;s__uncultured Bacteroides sp. SMG1 | 5.53E-07 | 5.53E-07 | 3.24E-06 | 7.13E-07 | 0.019 |

Numbers in blue indicate *Bacteroides* species (n = 47) significantly enriched during hibernation; numbers in red indicate *Bacteroides* species (n = 10) significantly enriched in the active state.

**Table S13.** Comparison of the relative abundance of mucin oligosaccharide-degrading bacteria in hibernating versus active Chinese alligators. The relative abundance was determined by faecal the shotgun metagenomic sequencing.

| Bacterial species | Relative abundance (%) | | | |  | References |
| --- | --- | --- | --- | --- | --- | --- |
|  | Hibernation | S.E (H) | Active | S.E (A) | P value |  |
| s__Bacteroides thetaiotaomicron CAG:40 | 0.1444 | 0.0225 | 0.0239 | 0.0173 | **0.008** | (Tailford et al., 2015, Sonnenburg 2005) |
| s__Bacteroides thetaiotaomicron | 0.1247 | 0.0189 | 0.0710 | 0.0459 | 0.321 |  |
| s__Bacteroides vulgatus | 0.1289 | 0.0239 | 0.0335 | 0.0150 | **0.014** | (Xu et al., 2007) |
| s__Bacteroides fragilis | 2.3263 | 0.4459 | 4.7129 | 4.3739 | 0.640 |  |
| s__Bacteroides eggerthii | 0.0820 | 0.0231 | 0.0291 | 0.0082 | **0.044** | (Adamberg et al., 2018) |
| s__Bacteroides caccae | 0.1579 | 0.0178 | 0.0583 | 0.0310 | **0.021** | (Tailford et al., 2015) |
| s__Akkermansia muciniphila | 0.0003 | 0.0003 | 0.0029 | 0.0008 | **0.017** | (Derrien et al., 2004) |
| s__Akkermansia muciniphila CAG:154 | 0.0001 | 0.0000 | 0.0053 | 0.0013 | **0.009** |  |
| s__Akkermansia sp. CAG:344 | 0.0006 | 0.0005 | 0.0161 | 0.0016 | **0.001** |  |
| s__Ruminococcus gnavus CAG:126 | 0.0003 | 0.0002 | 0.0000 | 0.0000 | 0.333 | (Crost et al., 2013) |
| s__Ruminococcus torques | 0.0017 | 0.0012 | 0.0003 | 0.0002 | 0.277 | (Png et al., 2010) |
| s__Bifidobacterium bifidum | 0.0001 | 0.0001 | 0.0001 | 0.0001 | 0.724 | (Turroni et al., 2010) |
| s__Bifidobacterium longum | 0.0003 | 0.0003 | 0.0003 | 0.0001 | 0.958 | (Patricia et al., 2008) |

| **Table S13 References:** |
| --- |

Tailford LE, Crost EH, Devon K, Nathalie J. 2015. Mucin glycan foraging in the human gut microbiome. Front Genet 6:81.

Sonnenburg JL, Xu J, Leip DD, Chen CH, Westover BP, Weatherford J, Buhler JD, Gordon JI. 2005. Glycan Foraging in Vivo by an Intestine-Adapted Bacterial Symbiont. Science 307:1955-1959.

Xu J, Mahowald MA, Ley RE, Lozupone CA, Hamady M, Martens EC, Henrissat B, Coutinho PM, Minx P, Latreille P. 2007. Evolution of Symbiotic Bacteria in the Distal Human Intestine. Plos Biol 5:e156-e156.

Adamberg K, Kolk K, Jaagura M, Vilu R, Adamberg S. 2018. The composition and metabolism of faecal microbiota is specifically modulated by different dietary polysaccharides and mucin: an isothermal microcalorimetry study. Beneficial Microbes 9:21-34.

Derrien M, Vaughan EE, Plugge CM, de Vos WM. 2004. Akkermansia muciniphila gen. nov., sp. nov., a human intestinal mucin-degrading bacterium. Int J Syst Evol Microbiol 54:1469-1476.

Crost EH, Tailford LE, Le GG, Fons M, Henrissat B, Juge N. 2013. Utilisation of Mucin Glycans by the Human Gut Symbiont Ruminococcus gnavus Is Strain-Dependent. Plos One 8:e76341.

Png CW, Lindén SK, Gilshenan KS, Zoetendal EG, McSweeney CS, Sly LI, McGuckin MA, Florin TH. 2010. Mucolytic bacteria with increased prevalence in IBD mucosa augment in vitro utilization of mucin by other bacteria. Am J Gastroenterol 105:2420-2428.

Turroni F, Bottacini F, Foroni E, Mulder I, Kim JH, Zomer A, Sánchez B, Bidossi A, Ferrarini A, Giubellini V. 2011. Genome analysis of Bifidobacterium bifidum PRL2010 reveals metabolic pathways for host-derived glycan foraging. Proc Natl Acad Sci USA 107:19514-9.

Patricia Ruas-Madiedo MGMF-GCGdlR-GAM. 2008. Mucin Degradation by Bifidobacterium Strains Isolated from the Human Intestinal Microbiota. Appl Environ Microb 74:1936-1940.

**Table S14.** The number of CAZyme families identified in faecal samples from hibernating versus active Chinese alligator

|  | GH (glycoside hydrolase) | CBM (carbohydrate binding module) | PL (polysaccharide lyase) | CE (carbohydrate esterase) | GT (glycosyl transferase) | Total |
| --- | --- | --- | --- | --- | --- | --- |
| Total | 93 | 36 | 15 | 11 | 38 | 193 |
| Hibernation | 86 (19) | 31 (2) | 13 (4) | 11 (2) | 35 (3) | 176 (30) |
| Active | 90 (5) | 34 (2) | 14 (0) | 11 (0) | 37 (0) | 186 (7) |

Numbers in brackets mean the number of significantly enriched CAZymes families during different phases.

**Table S15.** Relative abundance of significantly differentially enriched CAZyme families in hibernating vs. active Chinese alligators

| CAZyme families | Relative abundance (%) | | | | P value | Brief enzymatic characterization in CAZy database (Lombard, et al., 2014) |
| --- | --- | --- | --- | --- | --- | --- |
|  | Hibernation | S.E (H) | Active | S.E (A) |  |  |
| CBM14 | 0 | 0 | 2.03E-06 | 4.76E-07 | 0.014 | chitin binding |
| CBM9 | 6.42E-05 | 5.96E-06 | 0.00020 | 1.66E-05 | 0.004 | cellulose-binding |
| GH10 | 1.66E-05 | 8.07E-06 | 0.00012 | 1.36E-05 | 0.004 | endo-1,4-β-xylanase, et al., |
| GH133 | 1.20E-05 | 7.58E-06 | 0.00019 | 1.11E-05 | 0.000 | amylo-α-1,6-glucosidase, et al., |
| GH24 | 9.02E-05 | 3.81E-05 | 0.00080 | 0.00026 | 0.043 | lysozyme, et al., |
| GH38 | 7.25E-05 | 1.10E-05 | 0.00027 | 2.02E-05 | 0.003 | α-mannosidase, et al., |
| GH77 | 0.00032 | 1.08E-05 | 0.00037 | 9.81E-06 | 0.017 | α-mannosidase, et al., |
| **CBM32** | 0.00185 | 0.00027 | 0.00080 | 0.00020 | 0.033 | galactose, lactose binding/ recognize mucus polysaccharides |
| **CBM51** | 0.00126 | 0.00022 | 0.00041 | 2.65E-05 | 0.019 | galactose binding/ recognize mucin mucus polysaccharides |
| CE1 | 0.00072 | 0.00013 | 0.00022 | 2.83E-05 | 0.018 | acetyl xylan esterase; cinnamoyl esterase, et al., |
| **CE9** | 0.00062 | 1.14E-05 | 0.00035 | 1.93E-05 | 0.001 | N-acetylglucosamine 6-phosphate deacetylase |
| **GH111** | 3.12E-06 | 1.08E-06 | 3.22E-08 | 3.22E-08 | 0.038 | endo-β-N-acetylglucosaminidase, et al., |
| GH117 | 0.00076 | 9.80E-05 | 0.00027 | 9.00E-05 | 0.021 | α-1,3-L-neoagarooligosaccharide hydrolase, et al., |
| GH13 | 0.00305 | 0.00017 | 0.00185 | 9.16E-05 | 0.005 | α-amylase; pullulanase, et al., |
| **GH18** | 0.00109 | 7.36E-05 | 0.00074 | 3.09E-05 | 0.013 | endo-β-N-acetylglucosaminidase, et al., |
| **GH20** | 0.00347 | 0.00032 | 0.00227 | 0.00035 | 0.049 | β-hexosaminidase;β-1,6-N-acetylglucosaminidase; β-6-SO3-N-acetylglucosaminidase, et al., |
| GH23 | 0.00186 | 9.17E-05 | 0.00089 | 7.37E-05 | 0.003 | peptidoglycan lyase; chitinase, et al., |
| **GH30** | 0.00076 | 0.00011 | 0.00011 | 5.94E-05 | 0.008 | β-glucosidase; β-xylosidase; β-fucosidase, et al., |
| **GH36** | 0.00144 | 0.00013 | 0.00062 | 0.00023 | 0.030 | α-galactosidase; α-N-acetylgalactosaminidase, |
| GH37 | 1.38E-05 | 4.73E-06 | 8.83E-07 | 8.83E-07 | 0.044 | α,α-trehalase, et al., |

(Continued Table S15)

| CAZyme families | Relative abundance (%) | | | | P value | Brief enzymatic characterization in CAZy database (Lombard, et al., 2014) |
| --- | --- | --- | --- | --- | --- | --- |
|  | Hibernation | S.E (H) | Active | S.E (A) |  |  |
| **GH42** | 0.00016 | 1.18E-05 | 1.82E-05 | 3.15E-06 | 0.001 | β-galactosidase; α-L-arabinopyranosidase, et al., |
| GH53 | 0.00024 | 2.78E-05 | 7.08E-05 | 4.12E-06 | 0.006 | endo-β-1,4-galactanase, et al., |
| **GH57** | 0.00026 | 4.27E-05 | 0.00011 | 8.00E-06 | 0.024 | α-amylase; α-galactosidase, et al., |
| GH65 | 0.00092 | 0.00015 | 0.00018 | 9.87E-05 | 0.015 | α,α-trehalase; maltose phosphorylase, et al., |
| GH72 | 0.00036 | 3.21E-05 | 0.00022 | 3.35E-05 | 0.030 | β-1,3-glucanosyltransglycosylase, et al., |
| **GH84** | 0.00074 | 9.45E-05 | 0.00039 | 9.02E-06 | 0.022 | N-acetyl β-glucosaminidase; 3-O-(GlcNAc)-L-Ser/Thr β-N-acetylglucosaminidase, et al., |
| GH88 | 0.00101 | 0.00016 | 0.00041 | 6.18E-05 | 0.023 | d-4,5-unsaturated β-glucuronyl hydrolase, et al., |
| **GH89** | 0.00085 | 0.00016 | 0.00030 | 5.72E-05 | 0.026 | α-N-acetylglucosaminidase, et al., |
| **GH9** | 0.00036 | 2.41E-05 | 0.00014 | 2.85E-05 | 0.007 | endoglucanase;β-glucosidase, et al., |
| **GH95** | 0.00266 | 0.00044 | 0.00074 | 0.00039 | 0.027 | α-L-fucosidase; α-1,2-L-fucosidase; α-L-galactosidase, et al., |
| GT3 | 0.00034 | 4.79E-05 | 0.00012 | 1.34E-05 | 0.013 | glycogen synthase, et al., |
| GT47 | 0.00034 | 1.17E-05 | 0.00020 | 2.55E-06 | 0.002 | heparan β-glucuronyltransferase;heparan synthase |
| GT51 | 0.00182 | 6.26E-05 | 0.00103 | 0.00016 | 0.012 | murein polymerase, et al., |
| PL12 | 0.00038 | 7.10E-05 | 0.00017 | 3.60E-05 | 0.044 | heparin-sulfate lyase, et al., |
| PL13 | 0.00032 | 4.68E-05 | 4.89E-05 | 1.12E-05 | 0.007 | heparin lyase, et al., |
| PL15 | 0.00037 | 3.90E-05 | 0.00011 | 1.70E-05 | 0.006 | oligo-alginate lyase; alginate lyase, et al., |
| PL8 | 0.00122 | 0.00017 | 0.00061 | 5.63E-05 | 0.025 | hyaluronate lyase; chondroitin AC lyase, et al., |

Letters in blue indicate CAZyme families significantly enriched during hibernation; red indicates that CAZyme families significantly enriched during active phase. The families in bold are involved in binding and degradation of mucin oligosaccharides. Abbreviation: A, active phase; H, hibernation phase.

**Table S15 Reference:** Lombard, V., Golaconda Ramulu, H., Drula, E., Coutinho, P.M. & Henrissat, B. 2014. The carbohydrate-active enzymes database (CAZy) in 2013. Nucleic Acids Res, 42, D490-D495.

**Table S16.** Summary of percentage composition (%) of the four representative and most abundant bacterial phyla in fecal samples of different vertebrates

| Host Species | Firmicutes | Proteobacteria | Fusobacteria | Bacteroidetes | Feeding habits | Classification | References |
| --- | --- | --- | --- | --- | --- | --- | --- |
| Chinese alligator (Hibernation) | 9.84 | 30.69 | 0.89 | 57.95 | Carnivorous | Reptile | **This study** |
| Chinese alligator (Active) | 15.39 | 30.28 | 35.64 | 18.29 | Carnivorous |  |  |
| Alligator mississippiensis | 16.70 | 2.70 | 69.60 | 5.00 | Carnivorous |  | (Keenan et al., 2013) |
| Cheetah1 | 56.20 | 4.20 | 18.10 | 5.80 | Carnivorous | Mammalia | （Menke, et al., 2014） |
| Black-backed jackal | 40.50 | 6.90 | 21.80 | 26.10 | Carnivorous |  | （Menke, et al., 2014） |
| Cheetah2 | 68.50 | 6.30 | 18.40 | 6.50 | Carnivorous |  | （Uddin, et al., 2017） |
| Wolf | 60.00 | 9.20 | 9.20 | 16.90 | Carnivorous |  | （Zhang, et al., 2010） |
| Tasmanian devil | 53.50 | 18.60 | 13.80 | 1.20 | Carnivorous |  | （Cheng, et al., 2015） |
| Dog | 14-28 | 5-7 | 23-40 | 31-34 | Carnivorous |  | （Suchodolski, et al., 2010） |
| Antarctic seals | 41.50 | 17.00 | 25.60 | 14.10 | Carnivorous |  | （Nelson, et al., 2013） |
| Harbor seals | 19-43 | 5-17 | 18-32 | 22-36 | Carnivorous |  | （Daniela, et al., 2016） |
| Dolphin | 56.00 | 27.00 | 4.00 | 3.00 | Carnivorous |  | （Soverini, et al., 2016） |
| Black vulture | 67.10 | 11.20 | 21.20 |  | Carnivorous | Bird | （Roggenbuck, et al., 2014） |
| Turkey vulture | 60.80 | 5.40 | 31.10 |  | Carnivorous |  |  |
| Red-crowned crane | 62.90 | 29.90 | 9.60 |  | Carnivorous |  | （Xie, et al., 2016） |
| Asian seabass | 15.30 | 48.80 | 7.30 | 8.20 | Carnivorous | Fish | （Xia, et al., 2014） |
| Asian seabass (under starvation) | 10.10 | 39.10 | 2.61 | 36.00 | Carnivorous |  |  |
| Channel catfish | 0.05 | 3.66 | 94.92 | 0.15 | Carnivorous |  | （Larsen, et al., 2014） |
| Largemouth bass | 1.32 | 7.84 | 90.56 | 0.06 | Carnivorous |  |  |
| Bluegill | 0.58 | 15.85 | 82.60 | 0.56 | Carnivorous |  |  |

(Continued Table S16)

| Host Species | Firmicutes | Proteobacteria | Fusobacteria | Bacteroidetes | Feeding habits | Classification | References |
| --- | --- | --- | --- | --- | --- | --- | --- |
| Nonhibernating tree frog (Winter) | 42.94 | 14.98 | 4.67 | 19.07 | Insectivorous | Amphibian | （Weng, et al., 2016） |
| Artificially hibernating frog (4°C) | 13.26 | 57.82 | 0.23 | 17.63 | Insectivorous |  |  |
| Bar-headed geese (wild) | 83.20 | 11.76 |  | 0.86 | Herbivorous | Bird | （Wang, et al., 2016） |
| Bar-headed geese (capitive) | 51.63 | 5.52 |  | 38.41 | Herbivorous |  |  |
| Anolis lizards | 60.75 | 20.56 |  | 13.18 | Herbivorous | Reptile | （Ren, et al., 2016） |
| Turtles | 44.80 | 1.10 |  | 46.60 | Herbivorous |  | （Campos, et al., 2018） |
| Green turtles | 47.59 | 18.97 | 4.47 | 26.15 | Herbivorous |  | （Ahasan, et al., 2017） |
| Land iguana | 63.90 | 1.40 | 0.60 | 4.20 | Herbivorous |  | （Pei-Ying, et al., 2011） |
| Marine iguana | 75.10 | 0.60 |  | 8.20 | Herbivorous |  |  |
| Grass carp (high protein–low fibre) | 7.21 | 3.58 | 76.07 | 7.36 | Herbivorous | Fish | （Hao, et al., 2017） |
| Grass carp (high fibre–low protein) | 54.48 | 4.62 | 5.98 | 10.09 | Herbivorous |  |  |
| Grass carp (intermediate) | 44.69 | 19.67 | 4.12 | 16.91 | Herbivorous |  |  |
| Brown bear (winter) | 24.98 | 31.01 | 1.44 | 39.91 | Omnivorous | Mammalia | （Sommer, et al., 2016） |
| Brown bear (summer) | 39.29 | 39.56 | 1.12 | 6.05 | Omnivorous |  |  |
| Andean bear (wild) | 18.56 | 51.77 | 0.02 | 11.30 | Omnivorous |  | （Borbóngarcía, et al., 2017） |
| Ground squirrel (Summer) | 65.50 | 1.50 |  | 21.60 | Omnivorous |  | （Carey, et al., 2013） |
| Ground squirrel (Late Winter) | 21.80 | 4.00 |  | 52.20 | Omnivorous |  |  |
| Wild wood mouse | 52.10 | 8.20 | 0.01 | 37.00 | Omnivorous |  | （Maurice, et al., 2015） |
| Lizard (L. parvus) | 38.75 | 8.72 |  | 45.33 | Omnivorous | Reptile | （Kohl, et al., 2017） |
| Lizard (L. ruibali) | 33.15 | 9.74 |  | 43.17 | Omnivorous |  |  |
| Crocodile lizard | 2.60 | 47.90 |  | 32.10 | Omnivorous |  | （Jiang, et al., 2017） |
| Surgeonfishes | 67.80 | 18.40 | 4.20 | 2.20 | Omnivorous | Fish | （Miyake, et al., 2015） |

**Table S16 References:**

Keenan SW, Engel AS, Elsey RM. 2013. The alligator gut microbiome and implications for archosaur symbioses. Sci Rep 3.

Menke S, Meier M, Melzheimer J, Mfune JKE, Heinrich S, Thalwitzer S, Wachter B, Sommer S. 2014. Oligotyping reveals differences between gut microbiomes of free-ranging sympatric Namibian carnivores (*Acinonyx jubatus*, *Canis mesomelas*) on a bacterial species-like level. Front Microbiol 5:1-12.

Uddin W, Menke S, Melzheimer J, Thalwitzer S, Heinrich S, Wachter B, Sommer S. 2017. Gut microbiomes of free-ranging and captive Namibian cheetahs: diversity, putative functions, and occurrence of potential pathogens. Mol Ecol 26 (20).

Zhang H, Chen L. 2010. Phylogenetic analysis of 16S rRNA gene sequences reveals distal gut bacterial diversity in wild wolves (*Canis lupus*). Mol Biol Rep 37: 4013-4022.

Cheng YY, Fox S, Pemberton D, Hogg C, Papenfuss AT, Belov K. 2015. The Tasmanian devil microbiome-implications for conservation and management. Microbiome 3: 11.

Suchodolski JS, Camacho J, Steiner JM. 2010. Analysis of bacterial diversity in the canine duodenum, jejunum, ileum, and colon by comparative 16S rRNA gene analysis. FEMS Microbiol Ecol 66:567-578.

Nelson TM, Rogers TL, Carlini AR, Brown MV. 2013. Diet and phylogeny shape the gut microbiota of Antarctic seals: a comparison of wild and captive animals. Environ Microbiol 15:1132.

Daniela N, Herlemann DPR, Klaus J, Guido D, Heide SV. 2016. Comparative analysis of the fecal bacterial community of five harbor seals (*Phoca vitulina*). Microbiologyopen 5: 782-792.

Soverini M, Quercia S, Biancani B, Furlati S, Turroni S, Biagi E, Consolandi C, Peano C, Severgnini M, Rampelli S, Brigidi P, Candela M. 2016. The bottlenose dolphin (*Tursiops truncatus*) faecal microbiota. FEMS Microbiol Ecol 92:8.

Roggenbuck M, Schnell IB, Blom N, Baelum J, Bertelsen MF, Ponten TS, Sorensen SJ, Gilbert MTP, Graves GR, Hansen LH. 2014. The microbiome of New World vultures. Nat Commun 5:7.

Xie Y, Pu X, Hui W, Yu H, Giesy JP, Zhang Y, Mora MA, Zhang X. 2016. Effects of captivity and artificial breeding on microbiota in feces of the red-crowned crane (*Grus japonensis*). Sci Rep 6:33350.

Xia JH, Lin G, Fu GH, Wan ZY, Lee M, Wang L, Liu XJ, Yue GH. 2014. The intestinal microbiome of fish under starvation. Bmc Genomics 15:266.

Larsen AM, Mohammed HH, Arias CR. 2014. Characterization of the gut microbiota of three commercially valuable warmwater fish species. J Appl Microbiol 116: 1396-1404.

Weng CH, Yang YJ, Wang D. 2016. Functional analysis for gut microbes of the brown tree frog (*Polypedates megacephalus*) in artificial hibernation. Bmc Genomics 17: 1024.

Wang W, Zheng S, Sharshov K, Sun H, Yang F, Wang X, Li L, Xiao Z. 2016. Metagenomic profiling of gut microbial communities in both wild and artificially reared Bar-headed goose (*Anser indicus*). Microbiologyopen 6.

Ren TT, Kahrl AF, Wu M, Cox RM. 2016. Does adaptive radiation of a host lineage promote ecological diversity of its bacterial communities? A test using gut microbiota of Anolis lizards. Mol Ecol 25:4793-4804.

Campos P, Guivernau M, Prenafeta-Boldu FX, Cardona L. 2018. Fast acquisition of a polysaccharide fermenting gut microbiome by juvenile green turtles Chelonia mydas after settlement in coastal habitats. Microbiome 6: 11.

Ahasan MS, Waltzek TB, Huerlimann R, Ariel E. 2017. Fecal bacterial communities of wild-captured and stranded green turtles (*Chelonia mydas*) on the Great Barrier Reef. FEMS Microbiol Ecol 93: 11.

Pei-Ying Hong EWIKOCRIM. 2011. Phylogenetic analysis of the fecal microbial community in herbivorous land and marine iguanas of the Galápagos Islands using 16S rRNA-based pyrosequencing. ISME J 5:1461-70.

Hao YT, Wu SG, Jakovlic I, Zou H, Li WX, Wang GT. 2017. Impacts of diet on hindgut microbiota and short-chain fatty acids in grass carp (*Ctenopharyngodon idellus*). Aquac Res 48:5595-5605.

Sommer F, Ståhlman M, Ilkayeva O, Arnemo JM, Kindberg J, Josefsson J, Newgard CB, Fröbert O, Bäckhed F. 2016. The Gut Microbiota Modulates Energy Metabolism in the Hibernating Brown Bear Ursus arctos. Cell Rep 14:1655-1661.

Borbóngarcía A, Reyes A, Vivesflórez M, Caballero S. 2017. Captivity Shapes the Gut Microbiota of Andean Bears: Insights into Health Surveillance. Front Microbiol 8.

Carey HV, Walters WA, Knight R. 2013. Seasonal restructuring of the ground squirrel gut microbiota over the annual hibernation cycle. Am J Physiol-Regul Integr Comp Physiol 304:R33-R42.

Maurice CF, Knowles SC, Ladau J, Pollard KS, Fenton A, Pedersen AB, Turnbaugh PJ. 2015. Marked seasonal variation in the wild mouse gut microbiota. ISME J 9:2423-2434.

Kohl KD, Brun A, Magallanes M, Brinkerhoff J, Laspiur A, Acosta JC, Caviedes-Vidal E, Bordenstein SR. 2017. Gut microbial ecology of lizards: insights into diversity in the wild, effects of captivity, variation across gut regions and transmission. Mol Ecol 26:1175-1189.

Jiang HY, Ma JE, Li J, Zhang XJ, Li LM, He N, Liu HY, Luo SY, Wu ZJ, Han RC. 2017. Diets Alter the Gut Microbiome of Crocodile Lizards. Front Microbiol 8:2073.

Miyake S, Ngugi DK, Stingl U. 2015. Diet strongly influences the gut microbiota of surgeonfishes. Mol Ecol 24:656-72.

**Table S17.** Comparison of the relative abundance of protein-, peptide-, and AA-degrading taxa between hibernating and active Chinese alligators. The relative abundance was determined by faecal the shotgun metagenomic sequencing.

| Phylum | Genus | Species | Relative Abundance (%) | | | | P value | References |
| --- | --- | --- | --- | --- | --- | --- | --- | --- |
|  |  |  | Hibernation | S.E (H) | Active | S.E (A) |  |  |
| Fusobacteria | *Cetobacterium* | *C.somerae* | 0.8085 | 0.4290 | 33.9128 | 16.8175 | **0.04** | (Finegold et al., 2003, Tsuchiya et al., 2010) |
|  | *Fusobacterium* | *F.nucleatum* | 0.0017 | 0.0010 | 0.0339 | 0.0178 | 0.17 | (Dai et al., 2015) |
|  |  | *F.varium* | 0.0046 | 0.0025 | 0.0576 | 0.0294 | 0.17 | (Potrykus et al., 2008) |
| Proteobacteria | *Escherichia* | *E.coli* | 0.1867 | 0.1072 | 0.1354 | 0.0214 | 0.67 | (Dai et al., 2010) |
|  | *Klebsiella* | *K.pneumoniae* | 0.0308 | 0.0140 | 0.0133 | 0.0053 | 0.29 | (Dai et al., 2010) |
|  | *Campylobacter* | *C.jejuni* | 0.0025 | 0.0012 | 0.0034 | 0.0019 | 0.71 | (Dai et al., 2011) |
| Firmicutes | *Peptoclostridium* | *C.bifermentans* | 0.0023 | 0.0005 | 0.0565 | 0.0280 | 0.11 | (Dai et al., 2015, Smith et al., 1998) |
|  |  | *C.sticklandii* | 0.0028 | 0.0024 | 0.0024 | 0.0009 | 0.88 | (Dai et al., 2011) |
|  | *Clostridium* | *C.sporogenes* | 0.0011 | 0.0006 | 0.0063 | 0.0025 | 0.06 | (Dai et al., 2011, Allison et al., 1990) |
|  |  | *C.tunisiense* | 0.0026 | 0.0012 | 0.0264 | 0.0126 | 0.14 | (Zeibich et al., 2018, Thabet et al., 2004) |
|  |  | *C.tetanomorphum* | 0.0016 | 0.0006 | 0.0071 | 0.0028 | 0.11 | (Dai et al., 2011) |
|  |  | *C.perfringens* | 0.0074 | 0.0040 | 0.0856 | 0.0377 | **0.05** | (Schwab et al., 2011, Zentek et al., 2010) |
|  | *Megasphaera* | *M.elsdenii* | 0.0001 | 0.0001 | 0.0021 | 0.0006 | **0.02** | (Dai et al., 2010) |
|  | *Selenomonas* | *S.ruminantium* | 0.0005 | 0.0005 | 0.0134 | 0.0020 | **0.00** | (Dai et al., 2011) |
|  | *Acidaminococcus* | *A.fermentans* | 0.0000 | 0.0000 | 0.0002 | 0.0000 | **0.01** | (Dai et al., 2010) |
|  | *Staphylococcus* | *S.aureus* | 0.0004 | 0.0002 | 0.0014 | 0.0002 | **0.01** | (Soverini et al., 2016, Dai et al., 2015) |
|  | *Lactobacillus* | *L.paracasei* | 0.0000 | 0.0000 | 0.0001 | 0.0001 | 0.18 | (Dai et al., 2015) |
|  | *Butyrivibrio* | *B.fibrisolvens* | 0.0018 | 0.0005 | 0.0025 | 0.0003 | 0.10 | (Dai et al., 2011) |
|  | *Mitsuokella* |  | 0.0003 | 0.0002 | 0.0043 | 0.0005 | **0.00** | (Dai et al., 2010) |
| Bacteroidetes | *Prevotella* | *P.ruminicola* | 0.0005 | 0.0005 | 0.0028 | 0.0004 | **0.01** | (Dai et al., 2011) |
| Actinobacteria | *Bifidobacterium* | *B.dentium* | 0.0003 | 0.0003 | 0.0004 | 0.0002 | 0.77 | (Dai et al., 2015) |

**Table S17 References:**

| Finegold SM, Vaisanen M-L, Molitoris DR, Tomzynski TJ, Song Y, Liu C, Collins MD, Lawson PA. 2003. *Cetobacterium somerae* sp. nov. from human feces and emended description of the genus Cetobacterium. Syst Appl Microbiol 26:177-181.  Tsuchiya C, Sakata T, Sugita H. 2010. Novel ecological niche of *Cetobacterium somerae*, an anaerobic bacterium in the intestinal tracts of freshwater fish. Lett Appl Microbiol 46:43-48.  Dai Z, Wu Z, Hang S, Zhu W, Wu G. 2015. Amino acid metabolism in intestinal bacteria and its potential implications for mammalian reproduction. Mol Hum Reprod 21:389-409.  Potrykus J, White RS. 2008. Proteomic investigation of amino acid catabolism in the indigenous gut anaerobe *Fusobacterium varium*. Proteomics 8:2691-2703.  Dai ZL, Zhang J, Wu G, Zhu WY. 2010. Utilization of amino acids by bacteria from the pig small intestine. Amino Acids 39:1201-1215.  Dai ZL, Wu G, Zhu WY. 2011. Amino acid metabolism in intestinal bacteria: links between gut ecology and host health. Front Biosci-Landmrk 16:1768.  Dai Z, Wu Z, Hang S, Zhu W, Wu G. 2015. Amino acid metabolism in intestinal bacteria and its potential implications for mammalian reproduction. Mol Hum Reprod 21:389-409.  Smith EA, Macfarlane GT. 1998. Enumeration of amino acid fermenting bacteria in the human large intestine: effects of pH and starch on peptide metabolism and dissimilation of amino acids. FEMS Microbiol Ecol 25:355-368.  Allison C, Macfarlane GT. 1990. Regulation of protease production in Clostridium sporogenes. Appl Environ Microb 56:3485-3490.  Zeibich L, Schmidt O, Drake HL. 2018. Protein- and RNA-Enhanced Fermentation by Gut Microbiota of the Earthworm Lumbricus terrestris. Appl Environ Microb 84:17.  Thabet OB, Fardeau ML, Joulian C, Thomas P, Hamdi M, Garcia JL, Ollivier B. 2004. *Clostridium tunisiense* sp. nov., a new proteolytic, sulfur-reducing bacterium isolated from an olive mill wastewater contaminated by phosphogypse. Anaerobe 10:185-190.  Schwab C, Gänzle M. 2011. Comparative analysis of fecal microbiota and intestinal microbial metabolic activity in captive polar bears. Can J Microbiol 57:177-85.  Zentek J, Marquart B, Pietrzak T, Ballèvre O, Rochat F. 2010. Dietary effects on bifidobacteria and Clostridium perfringens in the canine intestinal tract. J Anim Physiol Anim Nutr 87:397-407.  Soverini M, Quercia S, Biancani B, Furlati S, Turroni S, Biagi E, Consolandi C, Peano C, Severgnini M, Rampelli S, Brigidi P, Candela M. 2016. The bottlenose dolphin (*Tursiops truncatus*) faecal microbiota. FEMS Microbiol Ecol 92:8. |
| --- |

**Table S18.** Differences in the relative abundance of archaeal taxa between hibernation and active state

| Taxa | Hibernation | S.E (H) | Active | S.E (A) | P value |
| --- | --- | --- | --- | --- | --- |
| f__Methanocorpusculaceae | 3.11E-06 | 1.21E-06 | 5.17E-05 | 7.13E-06 | **0.007** |
| g__Methanocorpusculum | 3.11E-06 | 1.21E-06 | 5.17E-05 | 7.13E-06 | **0.006** |
| f__Methanococcaceae | 2.64E-06 | 1.84E-06 | 3.39E-05 | 1.84E-05 | **0.008** |
| g__Methanococcus | 2.64E-06 | 1.84E-06 | 3.29E-05 | 1.80E-05 | 0.209 |
| g__Methanothermococcus | 0 | 0 | 1.01E-06 | 5.19E-07 | 0.114 |
| f__Methanoregulaceae | 1.03E-05 | 9.14E-06 | 1.67E-05 | 1.68E-06 | **0.014** |
| g__Methanoregula | 1.03E-05 | 9.14E-06 | 1.30E-05 | 3.63E-06 | 0.766 |
| g__Methanosphaerula | 0 | 0 | 3.71E-06 | 1.99E-06 | 0.154 |
| f__Methanocellaceae | 2.45E-06 | 2.03E-06 | 1.75E-05 | 8.76E-06 | **0.008** |
| g__Methanocella | 2.45E-06 | 2.03E-06 | 1.75E-05 | 8.76E-06 | 0.210 |
| f__Methanobacteriaceae | 1.66E-06 | 1.48E-06 | 2.58E-05 | 9.34E-06 | **0.034** |
| g__Methanobacterium | 1.50E-06 | 1.50E-06 | 1.48E-05 | 6.21E-06 | 0.067 |
| g__Methanobrevibacter | 1.59E-07 | 1.07E-07 | 1.10E-05 | 3.70E-06 | **0.026** |
| f__Methanospirillaceae | 1.09E-07 | 1.09E-07 | 1.48E-05 | 7.44E-06 | **0.008** |
| g__Methanospirillum | 1.09E-07 | 1.09E-07 | 1.48E-05 | 7.44E-06 | 0.105 |
| f__Methanosaetaceae | 5.87E-06 | 5.31E-06 | 9.96E-06 | 4.35E-06 | **0.014** |
| g__Methanosaeta | 5.87E-06 | 5.31E-06 | 9.96E-06 | 4.35E-06 | 0.601 |
| f__Methanomicrobiaceae | 1.64E-06 | 1.49E-06 | 1.68E-05 | 8.49E-06 | **0.008** |
| g__Methanoplanus | 1.60E-06 | 1.45E-06 | 1.05E-05 | 5.27E-06 | 0.219 |
| g__Methanoculleus | 4.10E-08 | 4.10E-08 | 5.10E-06 | 2.84E-06 | 0.182 |
| g__Methanofollis | 0 | 0 | 1.20E-06 | 8.09E-07 | 0.249 |
| f__Methanosarcinaceae | 4.63E-06 | 1.13E-06 | 1.95E-05 | 6.49E-06 | **0.007** |
|  |  |  |  |  |  |
| (Continued Table S18) |  |  |  |  |  |
| Taxa | Hibernation | S.E (H) | Active | S.E (A) | P value |
| g__Methanococcoides | 1.16E-06 | 1.08E-07 | 7.29E-06 | 1.88E-06 | **0.021** |
| g__Methanosarcina | 3.17E-06 | 1.08E-07 | 5.92E-06 | 1.88E-06 | 0.439 |
| g__Methanolobus | 0 | 0 | 4.16E-06 | 2.12E-06 | 0.111 |
| f__Methanosarcinaceae | 4.63E-06 | 1.13E-06 | 1.95E-05 | 6.49E-06 | **0.007** |
| g__Methanomethylovorans | 3.72E-08 | 3.72E-08 | 7.23E-07 | 7.23E-07 | 0.470 |
| f__Halobacteriaceae | 8.48E-07 | 6.33E-07 | 8.08E-06 | 2.69E-06 | **0.031** |
| g__Natronorubrum | 8.48E-07 | 6.33E-07 | 2.72E-06 | 1.36E-06 | 0.294 |
| g__Halarchaeum | 0 | 0 | 2.45E-06 | 1.23E-06 | 0.084 |
| g__Salinarchaeum | 0 | 0 | 2.01E-06 | 1.02E-06 | 0.109 |
| f__Thermococcaceae | 6.73E-07 | 3.95E-07 | 6.32E-05 | 3.33E-05 | **0.008** |
| g__Thermococcus | 6.26E-08 | 3.23E-08 | 1.70E-06 | 9.54E-07 | 0.201 |
| g__Pyrococcus | 6.10E-07 | 3.96E-07 | 0 | 0 | 0.237 |
| f__Thermoplasmataceae;g__Thermoplasma | 1.67E-06 | 9.77E-07 | 1.11E-06 | 6.30E-07 | 0.645 |
| f__Candidatus Methanoperedenaceae; g__Candidatus Methanoperedens | 6.26E-07 | 6.26E-07 | 8.44E-06 | 4.38E-06 | 0.188 |
| f__Unclassified;g__Methanomassiliicoccus | 3.11E-06 | 1.62E-06 | 7.57E-06 | 7.57E-06 | 0.607 |
| f__Sulfolobaceae;g__Acidianus | 6.61E-06 | 6.53E-06 | 1.11E-06 | 8.51E-07 | 0.517 |
| f__Ferroplasmaceae;g__Ferroplasma | 1.75E-07 | 1.75E-07 | 9.47E-07 | 5.84E-07 | 0.289 |

Relative abundance was determined by fecal the shotgun metagenomic sequencing.

Numbers in bold denote a significant difference (P < 0.05). Abbreviations: F_, family; G_, genus.

**Table S19.** Microbial composition of different carnivorous animals at the kingdom level based on the shotgun metagenomic analysis

| Species | Bacteria | Eukaryota | Archaea | Viruses | Others | Sources |
| --- | --- | --- | --- | --- | --- | --- |
| Armadillo | 89.82% | 1.03% | ND | ND | 9.15% | (Muegge et al., 2011) |
| BushDog1 | 91.22% | 1.15% | ND | <0.0001% | 7.63% |  |
| Echidna | 87.79% | 1.29% | ND | 0.083% | 10.84% |  |
| Hyena2 | 85.01% | 2.35% | 0.01% | ND | 12.63% |  |
| Lion1 | 88.90% | 0.52% | ND | 0.038% | 10.54% |  |
| Lion2 | 92.14% | 1.05% | ND | 0.063% | 6.75% |  |
| Polar Bear2 | 91.36% | 0.38% | <0.0001% | 0.31% | 7.96% |  |
| Chinese alligator (Hibernation) | 97.00% | 0.01% | 0.01% | 0.04% | 2.93% | This study |
| Chinese alligator (Active) | 86.70% | 0.03% | 0.04% | **4.03%** | 9.21% |  |

ND means non-detectable

**Table S19 References:**

Muegge BD, Kuczynski J, Knights D, Clemente JC, González A, Fontana L, Henrissat B, Knight R, Gordon JI. 2011. Diet drives convergence in gut microbiome functions across mammalian phylogeny and within humans. Science 332:970-974.

**Table S20.** Comparison of opportunistic pathogens identified at the genus level from faecal samples between hibernating versus active Chinese alligator by the shotgun metagenomic sequencing

| Bacterial genus | Hibernation | S.E (H) | Active | S.E (A) | P value |
| --- | --- | --- | --- | --- | --- |
| g__Edwardsiella | 0.01786 | 0.01764 | 0.01707 | 0.00272 | 0.956 |
| g__Shewanella | 0.02455 | 0.02317 | 0.00035 | 0.00015 | 0.351 |
| g__Citrobacter | 0.01613 | 0.00606 | 0.00672 | 0.00659 | 0.349 |
| g__Aeromonas | 0.10255 | 0.07932 | 0.00284 | 0.00101 | 0.291 |
| g__Treponema | 0.00014 | 4.04E-05 | 0.00031 | 1.90E-05 | **0.015** |
| g__Moraxella | 0 | 0 | 0.00002 | 0.00000 | **0.004** |
| g__Enterococcus | 0.00025 | 0.00011 | 0.00021 | 4.42E-05 | 0.729 |
| g__Helicobacter | 0.00232 | 0.00148 | 0.00082 | 0.00050 | 0.465 |
| g__Pseudomonas | 0.00075 | 0.00043 | 0.00038 | 2.35E-05 | 0.505 |
| g__Acinetobacter | 0.00021 | 3.71E-05 | 0.00032 | 0.00012 | 0.494 |
| g__Escherichia | 0.00224 | 0.00120 | 0.00151 | 0.00016 | 0.599 |
| g__Klebsiella | 0.00073 | 0.00034 | 0.00026 | 0.00012 | 0.285 |
| g__Yersinia | 0.00035 | 0.00023 | 0.00015 | 5.87E-07 | 0.487 |
| g__Photobacterium | 0.00013 | 8.44E-05 | 0.00027 | 0.00013 | 0.515 |
| g__Legionella | 3.83E-05 | 1.22E-05 | 3.38E-06 | 4.31E-07 | **0.028** |
| g__Haemophilus | 1.45E-05 | 1.33E-05 | 5.24E-05 | 1.41E-05 | 0.114 |
| g__Rhodopseudomonas | 3.71E-06 | 3.71E-06 | 2.03E-05 | 4.36E-06 | **0.027** |
| g__Mycoplasma | 9.35E-06 | 5.36E-06 | 1.68E-05 | 6.06E-06 | 0.485 |
| g__Mycobacterium | 4.72E-06 | 2.52E-06 | 3.64E-05 | 9.36E-06 | **0.020** |
| g__Bartonella | 1.69E-07 | 1.69E-07 | 8.78E-07 | 7.85E-07 | 0.499 |
| g__Arcobacter | 3.73E-05 | 2.21E-05 | 2.61E-05 | 1.24E-05 | 0.662 |
| g__Staphylococcus | 1.73E-05 | 5.10E-06 | 9.61E-05 | 2.64E-05 | **0.026** |
| g__Proteus | 4.47E-05 | 4.07E-05 | 0.00117 | 0.00113 | 0.439 |
| g__Erysipelothrix | 1.22E-05 | 2.01E-06 | 1.51E-05 | 1.51E-05 | 0.827 |
| g__Micrococcus | 0 | 0 | 7.26E-07 | 3.64E-07 | 0.088 |
| g__Rhodococcus | 6.42E-06 | 4.44E-06 | 2.85E-05 | 1.07E-05 | 0.139 |

Numbers in bold denote a significant difference (P < 0.05). Abbreviations: A, active state; H, hibernation; S.E, standard error. Letters in blue indicate pathogens genus significantly enriched during hibernation; red indicates that pathogens genus significantly enriched during active phase.

**Table S20 References:**

Uddin W, Menke S, Melzheimer J, Thalwitzer S, Heinrich S, Wachter B, Sommer S. 2017. Gut microbiomes of free-ranging and captive Namibian cheetahs: diversity, putative functions, and occurrence of potential pathogens. Mol Ecol 26:5515-5527.

Maurice CF, Knowles SC, Ladau J, Pollard KS, Fenton A, Pedersen AB, Turnbaugh PJ. 2015. Marked seasonal variation in the wild mouse gut microbiota. Isme Journal Multidisciplinary Journal of Microbial Ecology 9:2423-2434.

Xie Y, Pu X, Hui W, Yu H, Giesy JP, Zhang Y, Mora MA, Zhang X. 2016. Effects of captivity and artificial breeding on microbiota in feces of the red-crowned crane (*Grus japonensis*). Scientific Reports 6:33350.

Weng CH, Yang YJ, Wang D. 2016. Functional analysis for gut microbes of the brown tree frog (*Polypedates megacephalus*) in artificial hibernation. BMC Genomics 17:1024.

Ahasan MS, Waltzek TB, Huerlimann R, Ariel E. 2017. Fecal bacterial communities of wild-captured and stranded green turtles (*Chelonia mydas*) on the Great Barrier Reef. FEMS Microbiol Ecol 93:1-11.

Desai MS, Seekatz AM, Koropatkin NM, Kamada N, Hickey CA, Wolter M, Pudlo NA, Kitamoto S, Terrapon N, Muller A, Young VB, Henrissat B, Wilmes P, Stappenbeck TS, Nunez G, Martens EC. 2016. A dietary fiber-deprived gut microbiota degrades the colonic mucus barrier and enhances pathogen susceptibility. Cell 167:1339-1353.

Ahasan MS, Waltzek TB, Huerlimann R, Ariel E. 2018. Comparative analysis of gut bacterial communities of green turtles (*Chelonia mydas*) pre-hospitalization and post-rehabilitation by high-throughput sequencing of bacterial 16S rRNA gene. Microbiol Res 207:91-99.

Schwab C, Cristescu B, Northrup JM, Stenhouse GB, Gänzle M. 2011. Diet and environment shape fecal bacterial microbiota composition and enteric pathogen load of grizzly bears. Plos One 6:e27905.

Cariveau DP, Powell JE, Hauke K, Rachael W, Moran NA. 2014. Variation in gut microbial communities and its association with pathogen infection in wild bumble bees (*Bombus*). ISME J 8:2369-79.

Godoy-Vitorino F, Rodriguez-Hilario A, Alves AL, Goncalves F, Cabrera-Colon B, Mesquita CS, Soares-Castro P, Ferreira M, Marcalo A, Vingada J, Eira C, Santos PM. 2017. The microbiome of a striped dolphin (*Stenella coeruleoalba*) stranded in Portugal. Res Microbiol 168:85-93.

Cools P, Haelters J, Santiago GLDS, Claeys G, Boelens J, Leroux-Roels I, Vaneechoutte M, Deschaght P. 2013. Edwardsiella tarda sepsis in a live-stranded sperm whale (*Physeter macrocephalus*). Vet Microbiol 166:311-315.

Li TT, Li H, Gatesoupe FJ, She R, Lin Q, Yan XF, Li JB, Li XZ. 2017. Bacterial Signatures of "Red-Operculum" Disease in the Gut of Crucian Carp (*Carassius auratus*). Microb Ecol 74:510-521.

Keenan SW, Elsey RM. 2015. The Good, the Bad, and the Unknown: Microbial Symbioses of the American Alligator. Integr Comp Biol 55:972.
